# Design and Synthesis of Novel Helix Mimetics Based on the Covalent H-Bond Replacement and Amide Surrogate

**DOI:** 10.3390/molecules28020780

**Published:** 2023-01-12

**Authors:** Junyang Liu, Shoubin Tang, Jia-Lei Yan, Tao Ye

**Affiliations:** 1 Innovation Center of Marine Biotechnology and Pharmaceuticals, School of Biotechnology and Health Sciences, Wuyi University, Jiangmen 529020, China; 2 QianYan (Shenzhen) Pharmatech. Ltd., 5th Floor East, Building-3, Longcheng Industrial Park, Qinglin Roas West, Longgang District, Shenzhen 518172, China; 3 State Key Laboratory of Chemical Oncogenomics, Peking University Shenzhen Graduate School, University Town, Xili, Shenzhen 518055, China

**Keywords:** synthesis, helix mimetics, protein–protein interactions, PCNA

## Abstract

A novel hydrogen bond surrogate-based (HBS) α-helix mimetic was designed by the combination of covalent H-bond replacement and the use of an ether linkage to substitute an amide bond within a short peptide sequence. The new helix template could be placed in position other than the *N*-terminus of a short peptide, and the CD studies demonstrate that the template adopts stable conformations in aqueous buffer at exceptionally high temperatures.

## 1. Introduction

The α-helix is the most abundant secondary structural element in proteins and is found frequently at the interfaces of protein–protein interactions [1]. For example, transcriptional activator p53 contain a short α-helical sequence that mediates function by direct interaction with a receptor [2]. Stable isolated peptides with a defined short α-helical segment would be ideal inhibitors of macromolecular interactions. However, those with less than ~15 amino acid residues rarely adopt a defined conformation in isolation [3,4,5,6], and often lack the ability to fold into their bioactive conformation due to an entropic penalty for folding. New techniques for stabilizing short peptide helices may aid the design of inhibitors or mimics of protein function [7,8,9,10,11,12]. The head-to-backbone cross-linking strategy for helix stabilization includes salt bridges, metal chelates, and covalent cyclization methods such as disulfide and lactam bridges [13,14], hydrocarbon stapling [15,16], and hydrogen-bond surrogate (HBS) methods [17,18,19]. P53 can act as a tumor suppressor and induce cancer cell death, and the levels of p53 can be increased by blocking the p53–MDM2 interaction and reactivate the p53 function [20]. Thus, it is a promising therapeutic strategy for the treatment of cancers by designing and developing small-molecule inhibitors of the MDM2–p53 interaction [21,22]. We discovered that the CUL4A-DDB1-ROC1-L2DTL-PCNA ubiquitin E3 ligase complex interacts with MDM2 and p53 and regulates p53 polyubiquitination and proteolysis through MDM2. We also found that both p53 and MDM2 bind to PCNA directly through a conserved PIP box in p53 and MDM2 [23]. Based on these findings and our experience obtained from medicinal chemistry [24] and total synthesis of marine natural products [25,26,27,28,29,30,31,32], we initiated a chemical biology program aiming to develop inhibitors to block p53 degradation based on the PCNA binding motif, a helix-containing PIP-box of PCNA interaction proteins.

**Scheme 1:**
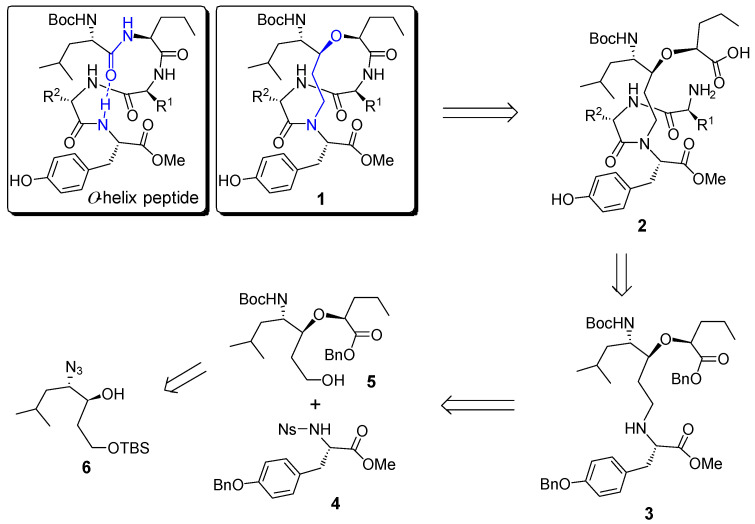
Structure of α-helix peptide and retrosynthetic analysis of helix template **1**.

Strategically placed covalent linkages of the type C = X − Y − N to replace the weak (*i*, *i* + 4) hydrogen bond, hydrogen-bond surrogates (HBS), have been shown to stabilize the helical conformations in short peptide sequences [17,18,19]. Modifications of the peptide backbone by the replacement of the hydrogen bond with hydrazine [17], carbon–carbon links [33], and thioether linkage [34] have been reported. This strategy provides peptides with increased target affinity and allows a stabilization of the α-helical conformation [9]. However, this approach seems limited to the *N*-terminal position of a short peptide, and could not be extended to our targeted system. In order to introduce the covalent H-bond replacement at an internal helical turn, we strategically employed an ether linkage to substitute an amide bond as well as replaced the corresponding (*i*, *i* + 4) hydrogen bond with a covalent ethylane bridge to afford a novel hydrogen bond surrogate-based (HBS) α-helix **1** (see Scheme 1). Notably, the new helix template (**1**) could be placed in position other than *N*-terminus of a short peptide. In connection with the previously mentioned chemical biology program, we selected the helix mimetic template **1** as a model system to explore the ability of the newly designed cyclopeptide to promote an *α*-helical conformation in aqueous solution.

The retrosynthetic analysis of the designed helix template (**1**) was shown in Scheme 1. The target molecule **1** could be obtained from a macrocyclization of the corresponding linear precursor **2**, which should be readily available from a coupling reaction of the advanced intermediate **3** with a suitable dipeptide. Further disconnection of **3** gave rise to the protected tyrosine derivative **4** and alcohol **5**, which is readily available from *β*-hydroxy azide **6**.

## 2. Results and Discussion

### 2.1. Synthesis of Helix Mimetic Template

**Scheme 2:**
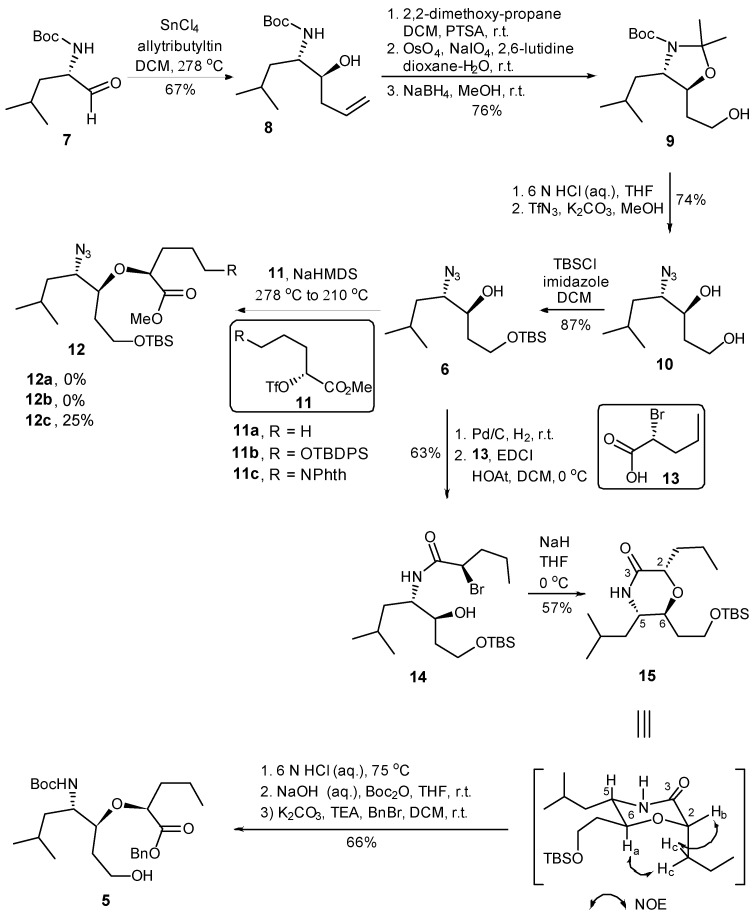
Synthesis of fragment **5**.

The synthesis commenced with the substrate controlled allylation of the known *α*-homochiral aldehyde **7** [35] (Scheme 2). Thus, the treatment of aldehyde **7** with allyltributyltin in the presence of SnCl_4_ at −78 °C afforded the *syn*- (**8**) and *anti*-homoallylic alcohols in a diastereomeric ratio of 9:1 [36,37]. Homoallylic alcohol **8** was converted into acetonide **9** in 76% overall yield via a three-step sequence including (i) oxazolidine formation with 2,2-dimethoxypropane/PTSA, (ii) the dihydroxylation of the terminal alkene with osmium tetroxide and 4-methylmorpholine *N*-oxide as co-oxidant, followed by sodium periodate cleavage of the diol, resulted in the corresponding aldehyde, and (iii) reduction in the resulting aldehyde with NaBH_4_ in methanol. The simultaneous removal of *N*-Boc and isopropylidene protective groups of **9** was realized under acidic conditions (6 M HCl in THF) to afford the corresponding amino diol, which was then converted into azide diol **10** in 74% yield via a diazo-transfer reaction with triflyl azide. The regioselective protection of the primary alcohol as its TBS ether afforded the key intermediate **6** in 87% yield.

We then addressed the formation of ether linkage of **12** so as to generate the required precursor **5** for further coupling reaction. Considering the nature of chiral triflate, also supported by literature precedents [38], we anticipated that the displacement of the triflate **11** by alkoxy anion derived from **6** could be achieved. Unfortunately, the attempted synthesis of the requisite ether **12** via the displacement reaction proved to be frustrating. All attempts to convert **6** to **12** by the reaction of the anion derived from the former with triflate **11**(**a**–**c**) [38], under a variety of reaction conditions provided no product or resulted in a much lower conversion (Scheme 2). In order to avoid more side-reaction derived from the slow intermolecular displacement process, we elected to construct the ether linkage via an intramolecular S_N_2 reaction, which would also avoid any ambiguity of the stereochemistry. Thus, the hydrogenation of the azide alcohol **6** over Pd/C gave the corresponding amine, which was condensed with (*R*)-2-bromopentanoic acid (**13**) employing EDCI to provide amide **14** in 63% yield over two steps. To our delight, upon the treatment of **14** with sodium hydride in THF, the intramolecular S_N_2 reaction proceeded smoothly to furnish the desired morphorlinone **15** as a single diastereomer in 57% yield. The inversion of the C-2 stereogenic center of **13** leading to the structure of **15** with the all-*S* stereochemical configurations was ascertained by NMR correlation and NOESY experiments. Morphorlinone **15** was then converted into the key intermediate **5** in 66% overall yield by a three-step sequence of straightforward transformations: (i) acid cleavage of TBS ether and the hydrolysis of amide to the corresponding amino acid; (ii) protection of the resultant amino group as its Boc carbamate; and (iii) protection of the carboxyl functionality as its benzyl ester.

**Scheme 3:**
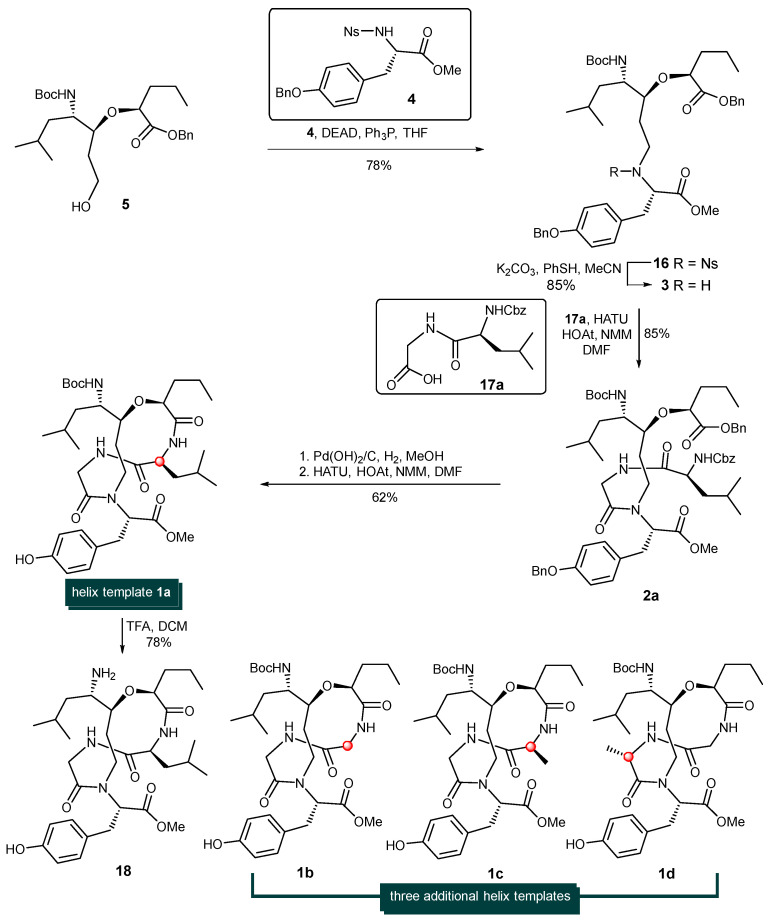
Synthesis of helix templates **1a**, **1b**, **1c**, **1d**.

With alcohol **5** at hand, attention was turned to the installation of the tyrosine units via the Fukuyama–Mitsunobu protocol and later-stage macrocyclization (Scheme 3). Thus, the treatment of sulfonamide-protected amine **4** [39,40,41] and alcohol **5** in the presence of diethylazodicarboxylate and triphenylphosphine afforded the corresponding alkylation adduct **16** in 78% yield. The nosyl group (Ns-) of **16** was then cleaved with Fukuyama’s thiophenol/K_2_CO_3_/CH_3_CN conditions [42], giving rise to the corresponding free secondary amine **3** in 85% yield. It is known that the coupling of a secondary amine and carboxylic acid using standard peptide coupling techniques is often a low-yielding process with certain difficulties. Gratifyingly, the HATU/HOAt-promoted coupling reaction between secondary amine **3** and dipeptide acid **17a** provided **2a** in 85% yield. The simultaneous removal of the benzyl ester, O-Bn ether and Cbz-protecting group was achieved by the hydrogenolysis of **2a** with Pd(OH)_2_ on carbon to produce the desired amino acid which was immediately activated by HATU/HOAT in the presence of *N*-methylmorpholine to afford cyclodepsipeptide **1a** in 62% yield. The steric hinderance of amine 3 significantly deterioate the reaction rate and yield of this peptide coupling step. Among all the tested coupling reagents such as PyAOP, DEPBT. HATU, BOPCl etc., HATU/HOAT condition was found with best results. The removal of the Boc- protecting group of **1a** with trifluoroacetic acid in CH_2_Cl_2_ produced the more hydrophilic template **18** in 78% yield (See Supplementary Materials).

By employing three additional dipeptides (Cbz-Gly-Gly-OH (**17b**), Cbz-L-Ala-Gly-OH (**17c**), and Cbz-Gly-L-Ala-OH (**17d**)) as coupling partners, helix-templates **1b**, **1c**, and **1d** were constructed via the identical procedure for the synthesis of **1a**. The structures of **1b**, **1c**, and **1d** are shown in Scheme 3.

### 2.2. CD Spectra of Helix Templates

**Figure 1:**
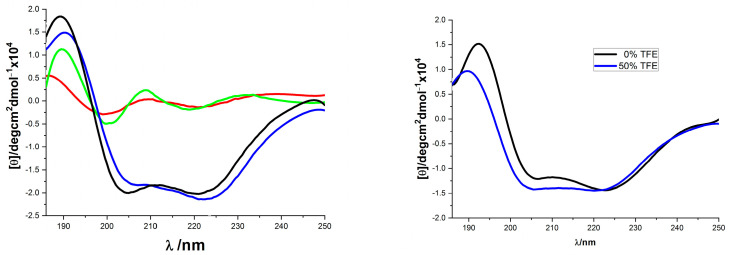
CD spectra of compounds **1** and **18**. ((**Left**) panel: **1a** (blue), **1b** (red), **1c** (black), and **1d** (green), all were recorded in 10 mM sodium phosphate buffer (pH 7.4); (**Right**) panel: **18** in 10 mM phosphate buffer pH 7.4 (black) and in 50% TFE (blue)).

**Figure 2:**
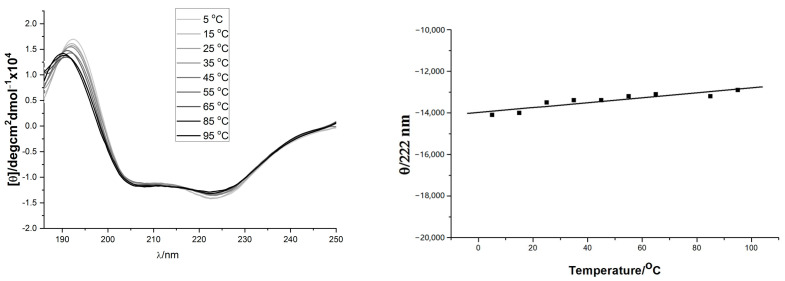
(**Left**) panel: Thermo-stability of **18** measured by CD spectra (from slim to thick line with 10 °C interval increase); (**Right**) panel: Temperature dependence of **18**’s mean peptide ellipticity at 222 nm (by linear fit).

The solution conformation of the helix-template **1a**–**d** was investigated by circular dichroism spectroscopy (Figure 1). All samples were measured at 10 mM concentration in pH 7.4 phosphate buffer. The CD spectra obtained for **1a** and **1c** were typical for the α-helical structure, showing the characteristic two molar ellipticity minima (λ = 222, 208 nm) and an ellipticity maximum (λ = 195 nm) [43,44]. However, the CD spectra of **1b** and **1d** showed almost no α-helical secondary structure (Figure 1, left panel). The loss of helicity in **1b** and **1d** relative to **1a** and **1c** indicated the stereogenic center and the substituent R1 of **1** (Scheme 1) are vital for the overall stability of helical conformation. Similar to **1a**, the CD spectra of **18** also showed two negative peaks at 208 and 222 nm wave trough and a positive value at 195 nm, which almost did not change spectral intensities by adding 2,2,2-trifluoroethanol (Figure 1, right panel). Previous thermal stability studies of HBS helices have shown that the conformations of these peptides remain remarkably consistent at high temperatures [19,33,45]. The thermal stabilities of **18** were investigated by monitoring the temperature-dependent change in the intensity of the 220 nm bands in the CD spectra (Figure 2). To our delight, we observed that the spectral lineshapes or intensities almost did not change along with a gradual increase in the temperature, even at 95 °C. Overall, the CD studies demonstrated that the template **18** adopts stable conformations.

## 3. Materials and Methods

### 3.1. General Experimental Details

All non-aqueous reactions were performed under an atmosphere of nitrogen or argon using oven-dried (120 °C) or flame-dried glassware under a N_2_ atmosphere. Commercially available reagents were used without further purification. All solvents were distilled prior to use: tetrahydrofuran (THF) from Na/benzophenone, dichloromethane (DCM), triethylamine (TEA) and dimethylformamide (DMF) were distilled from CaH_2_. Methanol (MeOH) was distilled under a N_2_ atmosphere from Mg/I_2_. ^1^H NMR and ^13^C NMR spectra were recorded in CDCl_3_ or MeOH-*d*_4_ on a Bruker Avance AV500 or Bruker Avance AV400 at 500 MHz (125 MHz) or 400 MHz (100 MHz), respectively. Chemical shifts are reported as *δ* values (ppm) referenced to either a tetramethylsilane (TMS) internal standard or the signals due to the solvent residual. Data for ^1^H NMR are reported as follows: chemical shift (*δ* ppm), multiplicity (s = singlet, br = broad, d = doublet, t = triplet, q = quartet, m = multiplet), coupling constant (Hz), integration. Mass spectra were measured on ABI Q-star Elite. Optical rotations were measured on a Perkin-Elmer 351 polarimeter at 589 nm with a 100 mm path length cell at 20 °C (reported as follows: concentration (*c* (in 1 g/100 mL), solvent). The reaction progress was checked on TLC plates. TLC was carried out using pre-coated sheets (Qingdao silica gel 60-F250, 0.2 mm) which, after development, were visualized under UV light at 254 nm, and/or staining in *p*-anisole, ninhydrin or phosphomolybdic acid solution followed by heating. Flash column chromatography was performed using the indicated solvents on Qingdao silica gel 60 (230–400 mesh ASTM). Yields refer to chromatographically purified compounds, unless otherwise stated.

CD spectroscopy studies: CD spectra were recorded on an Applied Photophysics Chirascan CD spectrometer equipped with a temperature controller using 0.1 cm path length cells. Experiments were performed at 20 °C using a 0.1 cm cell, at a scan speed of 100 nm/min from 250 to 186 nm. Spectral baselines were obtained under analogous conditions as that for the samples. All spectra were baseline subtracted, converted to a uniform scale of molar ellipticity, and smoothed using OriginPro 8.0 software. The samples were dissolved in 10 mM phosphate buffer, measured as pH 7.4. The raw CD data of **1a**–**1d** and **18** were recorded in raw ellipticity units. The concentrations of **1a**–**1d** and **18** were determined by quantitative RP-HPLC against a standard of known concentrations. All samples were between 20 and 70 μM, and the CD spectra were recorded in a range of concentrations to confirm that the sample aggregation did not occur. CD data were converted to mean the residue ellipticity, [θ] (deg∙cm^2^∙dmol^−1^), using the equation [θ] = *θ*/(10 × *c* × *l* × *n*), where *c* is the sample concentration (M) and *l* is the cell path length (cm), *n* is number of amino acid residues in the peptides (*n* = 4).

### 3.2. Procedures and Analytical Description of Compounds

*tert*-Butyl ((4*S*,5*S*)-5-hydroxy-2-methyloct-7-en-4-yl)carbamate **8**: SnCl_4_ (163 mL, 163 mmol, 1.0 M in CH_2_Cl_2_) was dropwise added to a solution of *N*-Boc-leucinal **7** (17.60 g, 81.6 mmol) in CH_2_Cl_2_ (380 mL) at −78 °C, 10 min later, allyltributyltin (35.20 g, 106 mmol) was dropwise added. The reaction mixture was stirred at −78 °C for 2 h and then quenched with saturated aqueous NH_4_Cl (50 mL). The organic layer was separated and dried with Na_2_SO_4_ and concentrated in vacuo. The residue was then dissolved in MeOH (400 mL) at 0 °C, after NaBH_4_ (5.40 g, 143 mmol) was added in three portions, the resulting mixture was vigorously stirred for 15 min and quenched with a saturated aqueous solution of NaHCO_3_ (100 mL). The reaction mixture was concentrated, and the residue was extracted with CH_2_Cl_2_ (100 mL × 3). The combined organic phase was dried over Na_2_SO_4_ and concentrated in vacuo. The residue was purified by column chromatography on silica gel to give compound **8** (14.10 g, 67%) as a colorless oil. [α]_D_^23^ = −33.8 (*c* 3.7, MeOH). ^1^H NMR (500 MHz, CDCl_3_) *δ* 5.84–5.77 (m, 1H), 5.10–5.06 (m, 2H), 4.80 (d, *J* = 9.5 Hz, 1H), 3.60–3.54 (m, 2H), 2.69 (brs, 1H), 2.28–2.23 (m, 1H), 2.19–2.13 (m, 1H), 1.65–1.59 (m, 1H), 1.49–1.36 (m, 11H), 0.90–0.86 (m, 6H) ppm. ^13^C NMR (125 MHz, CDCl_3_) *δ* 156.1, 134.7, 117.8, 78.9, 72.8, 51.8, 41.7, 38.9, 28.2, 24.7, 23.0, 22.0 ppm. HRMS (ESI, *m*/*z*) calculated for C_14_H_28_NO_3_^+^ [M+H]^+^: 258.2064, found 258.2058.

*tert*-Butyl (4*S*,5*S*)-5-(2-hydroxyethyl)-4-isobutyl-2,2-dimethyloxazolidine-3-carbo-xylate **9**: To a solution of **8** (5.00 g, 19.4 mmol) and 2,2-dimethoxypropane (3.6 mL, 29.3 mmol) in CH_2_Cl_2_ (150 mL) was added TsOH·H_2_O (85.0 mg, 0.45 mmol) at room temperature. The reaction mixture was stirred for 3 h at room temperature and then quenched with saturated aqueous NaHCO_3_ (50 mL) and extracted with additional CH_2_Cl_2_ (50 mL × 3). The combined organic layer was dried with Na_2_SO_4_. The residue was purified by column chromatography on silica gel to give the desired acetonide **8a** (5.25 g, 91%) as a colorless oil. [α]_D_^23^ = +10.7 (*c* 2.0, CHCl_3_). ^1^H NMR (500 MHz, CDCl_3_) *δ* 5.78–5.69 (m, 1H), 5.08–5.04 (m, 2H), 3.82–3.84 (m, H), 3.79–3.55 (m, 1H), 2.36–2.33 (m, 1H), 2.28–2.24 (m, 1H), 1.60–1.35 (m, 18H), 0.88 (s, 3H), 0.87 (s, 3H) ppm. ^13^C NMR (125 MHz, CDCl_3_) *δ* 151.7, 134.1, 117.6, 94.0, 80.3, 79.5, 60.0, 43.4, 40.3, 28.4, 25.4, 23.9, 21.2 ppm. HRMS (ESI, *mz*) calculated for C_17_H_32_NO_3_^+^ [M+H]^+^: 298.2377, found 298.2374.

The above acetonide **8a** (3.50 g, 11.7 mmol) was dissolved in dioxane–water (100 mL, 3:1), 2,6-lutidine (1.5 mL, 13.0 mmol), OsO_4_ (2.9 mL, 0.23 mmol, 0.079 M in *tert*-butanol) and NaIO_4_ (9.90 g, 46.0 mmol) were sequentially added to the solution at room temperature. The reaction mixture was stirred at room temperature and monitored by TLC. Upon all the starting acetonide being consumed, Na_2_S_2_O_3_ (30 mL, 60.0 mmol, 2 M in water) was added and the mixture was stirred for an additional 3 h to quench the reaction. The reaction mixture was extracted with ethyl acetate (50 mL × 4). The combined organic phase was washed with saturated aqueous NH_4_Cl (40 mL × 3), and brine (40 mL), dried over Na_2_SO_4_ and concentrated in vacuo. The residue was then dissolved in methanol (20 mL) at 0 °C, after NaBH_4_ (1.32 g, 35.0 mmol) was added, the reaction mixture was stirred at 0 °C for 15 min and then quenched by addition of a saturated aqueous solution of NH_4_Cl (50 mL). Volatiles were removed in vacuo, and the residue was extracted with ethyl acetate (50 mL × 3). The combined organic phase was washed with water (50 mL), brine (50 mL), dried over Na_2_SO_4_ and concentrated in vacuo. The purification of the residue by chromatography yielded **9** (2.97 g, 84%) as a colorless oil. [α]_D_^23^ = +4.96 (*c* 3.1, CHCl_3_). ^1^H NMR (500 MHz, CDCl_3_) *δ* 4.04–4.00 (m, 1H), 3.79–3.70 (m, 3H), 2.40 (s, 1H), 1.88–1.84 (m, 1H), 1.77–1.74 (m, 1H), 1.53–1.35 (m, 18H), 0.90 (s, 3H), 0.88 (s, 3H) ppm.^13^C NMR (125 MHz, CDCl_3_) *δ* 151.8, 94.0, 79.7, 79.2, 60.9, 60.2, 37.9, 43.5, 42.3, 37.9, 28.4, 25.5, 23.9, 21.4 ppm. HRMS (ESI, *m*/*z*) calculated for C_16_H_32_NO_4_^+^ [M+H]^+^: 302.2326, found: 302.2329.

(3*S*,4*S*)-4-Azido-6-methylheptane-1,3-diol **10**: A solution of HCl (5 mL, 6 M in water) was added to a solution of **9** (4.15 g, 13.7 mmol) in THF (20 mL). The reaction mixture was stirred at room temperature overnight. Volatiles were removed in vacuo, the residue was dissolved in MeOH (40 mL) and cooled to −15 °C. After K_2_CO_3_ (7.56 g, 54.8 mmol) and TfN_3_ (3.80 g, 22.0 mmol in CH_2_Cl_2_) were added, the reaction mixture was stirred at −15 °C for 1 h and allowed to warm to room temperature within 45 min. The reaction was then quenched with saturated aqueous NH_4_Cl (50 mL). Layers were separated, and the aqueous layer was extracted with CH_2_Cl_2_ (50 mL × 3). The combined organic layer was washed with brine (50 mL), dried over Na_2_SO_4_, and concentrated in vacuo. The residue was subjected to chromatographic purification, yielding compound **10** (1.89 g, 74%, two steps) as colorless oil. [α]_D_^23^ = −36.4 (*c* 1.6, CHCl_3_). ^1^H NMR (500 MHz, CDCl_3_) *δ* 3.93–3.89 (m, 1H), 3.85–3.79 (m, 2H), 3.60 (s, 1H), 3.32 (s, 1H), 3.20 (dt, *J* = 9.6, 4.1 Hz, 1H), 1.80–1.74 (m, 2H), 1.71–1.67 (m, 1H), 1.55 (ddd, *J* = 14.3, 10.0, 5.1 Hz, 1H), 1.33 (ddd, *J* = 13.5, 9.1, 4.1 Hz, 1H), 0.94–0.90 (m, 6H) ppm. ^13^C NMR (125 MHz, CDCl_3_) δ 73.3, 65.1, 60.5, 39.3, 35.5, 25.0, 23.1, 21.6 ppm. HRMS (ESI, *m*/*z*) calculated for C_8_H_18_N_3_O_2_^+^ [M+H]^+^: 188.1394, found: 188.1497.

(3*S*,4*S*)-4-Azido-1-((*tert*-butyldimethylsilyl)oxy)-6-methylheptan-3-ol **6**: *tert*-Butyl-dimethylsilyl chloride (2.80 g, 15.0 mmol) and imidazole (1.22 g, 18.0 mmol) were added to a solution of **10** (2.1 g, 11.2mmol) in DMF (10 mL) at room temperature. The reaction mixture was then stirred for 16 h before it was quenched by saturated sodium bicarbonate (20 mL) and extracted with ethyl acetate (50 mL × 3). The combined organic layers were washed with water (50 mL) and brine (50 mL), and dried over Na_2_SO_4_ and concentrated in vacuo. The residue was purified by flash chromatography to give compound **6** (3.93 g, 87%) as a colorless oil. [α]_D_^23^ = −6.5 (*c* 1.7, CHCl_3_). ^1^H NMR (500 MHz, CDCl_3_) *δ* 3.93–3.89 (m, 1H), 3.85–3.79 (m, 2H), 3.40 (d, *J* = 3.0 Hz, 1H), 3.18 (dt, *J* = 9.8, 4.0 Hz, 1H), 1.86–1.79 (m, 2H), 1.68–1.58 (m, 2H), 1.33 (ddd, *J* = 13.4, 9.0, 4.1 Hz, 1H), 0.96–0.93 (m, 6H), 0.89 (s, 9H), 0.08 (s, 6H) ppm. ^13^C NMR (125 MHz, CDCl_3_) *δ* 74.3, 64.5, 62.2, 39.1, 35.5, 25.8, 25.0, 23.2, 21.7, 18.1, −5.6, −5.6 ppm. HRMS (ESI, *m*/*z*) calculated for C_8_H_18_N_3_O_2_^+^ [M+H]^+^: 302.2258, found: 302.2267.

(*R*)-2-Bromo-N-((3*S*,4*S*)-1-((*tert*-butyldimethylsilyl)oxy)-3-hydroxy-6-methylheptan-4-yl)pentanamide **14**: Compound **6** (2.75 g, 9.1 mmol) was dissolved in MeOH (50 mL). After Pd(OH)_2_/C (0.23 g, 10% wt) was added, the reaction vessel was sealed and changed to a hydrogen atmosphere and stirred for 3 h. The catalyst was filtered off and the filtrate was dried over Na_2_SO_4_ and concentrated in vacuo to afford the crude amine, which was directly used in the next step coupling reaction. (2*R*)-2-Bromo-pentanoic acid **13** (2.7 g, 15.0 mmol) was dissolved in CH_2_Cl_2_ (15 mL), and pre-activated by EDCI (2.91 g, 15.1 mmol) and HOAt (2.28 g, 15.0 mmol) at room temperature for 0.5 h. A solution of the above crude amine in CH_2_Cl_2_ (20 mL) was added to the reaction mixture at 0 °C. The reaction mixture was then stirred overnight before it was quenched by saturated sodium bicarbonate (20 mL) and extracted with ethyl acetate (60 mL × 2). The combined organic layers were washed with water (20 mL), brine (20 mL), and evaporated in vacuo. The residue was purified by flash chromatography to give compound **14** (2.51 g, 63% two steps) as a colorless oil. [α]_D_^23^ = +0.5 (*c* 2.3, CHCl_3_). ^1^H NMR (500 MHz, CDCl_3_) *δ* 6.56 (d, *J* = 9.5 Hz, 1H), 4.28 (dd, *J* = 8.0, 5.7 Hz, 1H), 3.96–3.78 (m, 4H), 2.13–2.04 (m, 1H), 2.01–1.92 (m, 1H), 1.78–1.69 (m, 1H), 1.62–1.31 (m, 6H), 0.94–0.86 (m, 18H), 0.06 (s, 6H) ppm. ^13^C NMR (125 MHz, CDCl_3_) *δ* 168.8, 74.1, 62.8, 51.8, 51.1, 41.4, 37.7, 35.7, 25.7, 25.7, 24.7, 23.1, 22.1, 20.6, 18.0, 13.2, −5.6 (2C) ppm. HRMS (ESI, *m*/*z*) calculated for C_19_H_41_BrNO_3_Si^+^ [M+H]^+^: 438.2034, found: 438.2053.

(2*S*,5*S*,6*S*)-6-(2-((*tert*-Butyldimethylsilyl)oxy)ethyl)-5-isobutyl-2-propylmorpholin-3-one **15**: A solution of compound **14** (2.50 g, 5.70 mmol) in THF (10 mL) was dropwise added to a suspension of NaH (410 mg, 6.84 mmol, 60% dispensed in mineral oil) in THF (10 mL) at −20 °C. The reaction mixture was stirred for 1 h at −20 °C, then it was allowed to warm up slowly to 0 °C within 3 h and kept at 0 °C till TLC indicated the complete consumption of compound **14**. The reaction was quenched by the addition of a saturated aqueous solution of NH_4_Cl (20 mL) and extracted with ethyl acetate (50 mL × 3). The combined organic layer was washed with water (15 mL × 2) and brine (15 mL), dried over Na_2_SO_4_ and evaporated in vacuo. The residue was purified by flash chromatography to give compound **15** (1.17 g, 57%) as a colorless oil. [α]_D_^23^ = −25.5 (*c* 2.1, CHCl_3_). ^1^H NMR (500 MHz, CDCl_3_) *δ* 6.50 (s, 1H), 4.15–4.11 (m, 1H), 3.77–3.68 (m, 3H), 3.32–3.26 (m, 1H), 1.83–1.74 (m, 3H), 1.75–1.66 (m, 2H), 1.58–1.28 (m, 4H), 0.96–0.90 (m, 9H), 0.89 (s, 9H), 0.05 (s, 6H) ppm; ^13^CNMR (125 MHz, CDCl_3_) *δ* 172.1, 73.6, 68.8, 59.0, 54.0, 42.4, 34.2, 33.1, 25.8, 23.9, 23.6, 21.5, 18.9, 18.1, 13.6, −5.5 (2C) ppm. HRMS (ESI, *m*/*z*) calculated for C_19_H_40_NO_3_Si^+^ [M+H]^+^: 358.2772, found: 358.2761.

Benzyl (*S*)-2-(((3*S*,4*S*)-4-((tert-butoxycarbonyl)amino)-1-hydroxy-6-methylheptan-3-yl)oxy)pentanoate **5**: Compound **15** (0.57 g, 1.60 mmol) was heated at 75 °C in 6 M aqueous HCl (6 mL) for 3 h, after the reaction mixture was cooled to 0 °C, it was adjusted to pH 10–12 by the dropwise addition of 6 M aqueous solution of NaOH. Without further purification, the reaction mixture was diluted with THF (6 mL) and treated with Boc_2_O (0.45 mL, 2.10 mmol) at room temperature for 16 h. The reaction mixture was first extracted with diethyl ether (50 mL × 2). The organic extracts were discarded, while the aqueous layer was acidified to pH 1–2 at 0 °C with an 1 N aqueous solution of HCl and extracted with ethyl acetate (50 mL × 3). The combined organic layer was washed with brine (10 mL), dried over anhydrous Na_2_SO_4_, and concentrated in vacuo. The residue was dissolved in CH_2_Cl_2_ (20 mL) at 0 °C, after K_2_CO_3_ (0.22 g, 1.59 mmol), triethylamine (0.44 mL, 3.2 mmol) and BnBr (0.27 mL, 2.3 mmol) were sequentially added, the resulting mixture was gradually warmed to room temperature and stirred for 4 h. The reaction mixture was then quenched with water (2 mL) and extracted with ethyl acetate (50 mL × 3). The combined organic layer was washed with saturated NH_4_Cl (10 mL × 2) and brine (10 mL), dried over anhydrous Na_2_SO_4_ and concentrated in vacuo. The residue was purified by flash chromatography to give compound **5** (0.47 g, 66% over 3 steps) as a colorless oil. [α]_D_^23^ = −69.5 (*c* 0.86, CHCl_3_). ^1^H NMR (500 MHz, CDCl_3_) *δ* 7.36–7.33 (m, 5H), 5.19 (q, *J* = 12.2 Hz, 2H), 4.39 (d, *J* = 8.9 Hz, 1H), 4.25 (t, *J* = 5.7 Hz, 1H), 3.83–3.81 (m, 2H), 3.67 (dt, *J* = 11.1, 5.8 Hz, 1H), 3.65–3.55 (m, 1H), 3.28 (t, *J* = 6.2 Hz, 1H), 1.73–1.54 (m, 5H), 1.42–1.39 (m, 12H), 1.27–1.21 (m, 1H), 0.93–0.88 (m, 9H) ppm. ^13^C NMR (125 MHz, CDCl_3_) *δ* 174.1 155.6, 135.5, 128.6, 128.4 (2C), 79.4, 79.0, 66.9, 59.8, 49.7, 39.3, 35.5, 32.6, 28.4, 25.1, 23.5, 21.9, 18.7, 13.8 ppm. HRMS (ESI, *m*/*z*) calculated for C_25_H_42_NO_6_^+^ [M+H]^+^: 452.3007, found: 452.2994.

Benzyl (*S*)-2-(((4*S*,8*S*,9*S*)-4-(4-(benzyloxy)benzyl)-9-isobutyl-13,13-dimethyl-5-((4-nitrophenyl)sulfonyl)-3,11-dioxo-2,12-dioxa-5,10-diazatetradecan-8-yl)oxy)pentanoate **16**: Compounds **5** (0.85 g, 1.88 mmol), **4** (1.32 g, 2.82 mmol) and PPh_3_ (0.76 g, 2.90 mmol) were dissolved in THF (25 mL) at 0 °C, after DEAD (0.50 g, 2.90 mmol) was added, the reaction mixture was stirred for 6 h at room temperature. The reaction was quenched with water (20 mL). Volatiles were removed in vacuo, the aqueous residue was extracted with ethyl acetate (50 mL × 3). The combined organic layer was washed with water (30 mL) and brine (30 mL), dried over anhydrous Na_2_SO_4_ and evaporated in vacuo. The residue was purified by flash chromatography to give compound **16** (1.33 g, 78%) as a colorless oil. [α]_D_^23^ = −32.9 (*c* 0.80, CHCl_3_). ^1^H NMR (400 MHz, CDCl_3_) *δ* 7.81 (d, *J* = 7.7 Hz, 1H), 7.60 (d, *J* = 7.0 Hz, 1H), 7.53 (d, *J* = 8.1 Hz, 2H), 7.47–7.38 (m, 5H), 7.38–7.32 (m, 5H), 7.21 (d, *J* = 8.5 Hz, 2H), 6.89 (d, *J* = 8.5 Hz, 2H), 5.15 (s, 2H), 5.04 (s, 2H), 4.90 (t, *J* = 7.7 Hz, 1H), 4.48 (d, *J* = 9.3 Hz, 1H), 4.23–4.15 (m, 1H), 4.12 (t, *J* = 6.0 Hz, 1H), 3.82–3.72 (m, 2H), 3.58 (s, 3H), 3.39–3.29 (m, 2H), 2.97 (dd, *J* = 14.4, 8.5 Hz, 1H), 1.93–1.67 (m, 5H), 1.46 (s, 9H), 1.31–1.27 (m, 4H), 0.95–0.91 (m, 9H) ppm. ^13^C NMR (100 MHz, CDCl_3_) *δ* 172.9, 170.8, 157.7, 155.6, 148.3, 137.1, 135.8, 133.1, 133.0, 131.4, 130.9, 130.3, 128.6, 128.5, 128.4, 128.2, 128.1, 127.9, 127.5, 123.7, 114.9, 79.3, 79.3, 70.0, 66.6, 66.4, 61.7, 52.2, 49.6, 43.8, 39.7, 35.5, 35.4, 31.1, 28.4, 25.0, 23.5, 22.0, 18.5, 13.9 ppm. HRMS (ESI, *m*/*z*) calculated for C_48_H_62_N_3_O_12_S^+^ [M+H]^+^: 904.4049, found: 904.4076.

Benzyl (*S*)-2-(((4*S*,8*S*,9*S*)-4-(4-(benzyloxy)benzyl)-9-isobutyl-13,13-dimethyl-3,11-dioxo-2,12-dioxa-5,10-diazatetradecan-8-yl)oxy)pentanoate **3**: At 0 °C, K_2_CO_3_ (0.80 g 5.88 mmol) and PhSH (0.45 mL, 4.38 mmol) were added to a solution of **16** (1.32 g, 1.46 mmol) in CH_2_Cl_2_ (20 mL). The reaction mixture was then stirred at room temperature for 3 h, and then filtered through a pad of silica gel. The filtrate was concentrated in vacuo, and the residue was purified by flash chromatography to give compound **3** (0.89 g, 85%) as a colorless oil. [α]_D_^23^ = −26.8 (*c* 1.3, CHCl_3_). ^1^H NMR (500 MHz, CDCl_3_) *δ* 7.43–7.27 (m, 10H), 7.06 (d, *J* = 8.5 Hz, 2H), 6.87 (d, *J* = 8.5 Hz, 2H), 5.15 (s, 2H), 5.01 (s, 2H), 4.67 (d, *J* = 9.4 Hz, 1H), 4.03 (t, *J* = 6.2 Hz, 1H), 3.71 (dd, *J* = 14.5, 7.1 Hz, 1H), 3.62 (s, 3H), 3.46–3.37 (m, 2H), 2.88–2.77 (m, 2H), 2.69–2.61 (m, 1H), 2.55–2.47 (m, 1H), 1.70–1.51 (m, 5H), 1.42 (s, 9H), 1.40–1.23 (m, 4H), 0.93–0.88 (m, 9H) ppm. ^13^C NMR (125 MHz, CDCl_3_) δ 174.9, 172.9, 157.5, 155.6, 137.1, 135.6, 130.2, 129.8, 128.5, 128.4, 128.3, 128.3, 127.8, 127.3, 114.7, 79.7, 78.8, 78.2, 69.9, 66.3, 63.0, 51.4, 49.8, 44.5, 40.6, 38.7, 35.4, 31.0, 28.3, 28.3, 24.9, 23.1, 22.1, 18.5, 13.8 ppm. HRMS (ESI, *m*/*z*) calculated for C_42_H_59_N_2_O_8_^+^ [M+H]^+^: 719.4266, found: 719.4278.

Benzyl (5*S*,13*S*,15*S*)-10-((*S*)-3-(4-(benzyloxy)phenyl)-1-methoxy-1-oxopropan-2-yl)-13-((*S*)-1-((tert-butoxycarbonyl)amino)-3-methylbutyl)-5-isobutyl-3,6,9-trioxo-1-phenyl-15-propyl-2,14-dioxa-4,7,10-triazahexadecan-16-oate **2a**: Amine **3** (0.10 g, 0.14 mmol) and the dipeptide ((benzyloxy)carbonyl)-L-leucylglycine **17a** (0.18 g, 0.56 mmol) were dissolved in DMF (10 mL), after HATU (0.16 g, 0.42 mmol), HOAT (19 mg, 0.14 mmol) and NMM (88 μL, 0.80 mmol) were sequentially added at 0 °C, the reaction mixture was stirred at room temperature for 16 h. Volatiles were removed in vacuo, the residue was dissolved in ethyl acetate (150 mL) and successively washed with saturated aqueous NH_4_Cl (10 mL × 2), water (10 mL × 3) and brine (10 mL). The organic phase was then dried over Na_2_SO_4_ and evaporated in vacuo. The residue was purified by flash chromatography to give the desired product **2a** (121 mg, 85%). [α]_D_^23^ = −90.0 (*c* 1.4, CHCl_3_). ^1^H NMR (400 MHz, MeOH-*d*_4_) *δ* 7.45–7.28 (m, 15H), 7.15 (d, *J* = 8.4 Hz, 2H), 6.94 (d, *J* = 8.3 Hz, 2H), 5.23–5.07 (m, 4H), 5.03 (s, 2H), 4.30–4.13 (m, 2H), 3.96 (dd, *J* = 51.9, 16.9 Hz, 2H), 3.69 (s, 3H), 3.54–3.40 (m, 1H), 3.29–3.20 (m, 2H), 3.17–3.07 (m, 1H), 2.58 (d, *J* = 10.1 Hz, 1H), 1.79–1.58 (m, 7H), 1.44 (s, 9H), 1.38–1.21 (m, 5H), 0.99–0.88 (m, 15H) ppm. ^13^C NMR (100 MHz, MeOH-*d*_4_) *δ* 174.0, 173.1, 171.1, 168.8, 157.7, 157.1, 156.7, 137.3, 136.8, 135.9, 130.2, 130.2, 128.2, 128.1, 128.0, 127.6, 127.4, 127.4, 127.1, 114.7, 78.7, 78.4, 76.9, 69.7, 66.4, 66.2, 63.0, 53.6, 51.3, 46.4, 40.7, 37.7, 35.0, 33.3, 28.8, 27.4, 24.8, 24.5, 22.6, 22.1, 20.7, 20.4, 18.1, 12.8 ppm. HRMS (ESI, *m*/*z*) calculated for C_58_H_79_N_4_O_12_^+^ [M+H]^+^: 1023.5689, found: 1023.5667.

Methyl (*S*)-2-((2*S*,5*S*,13*S*)-13-((*S*)-1-((tert-butoxycarbonyl)amino)-3-methylbutyl)-5-isobutyl-3,6,9-trioxo-2-propyl-1-oxa-4,7,10-triazacyclotridecan-10-yl)-3-(4-hydroxyphen-yl)propanoate **1a**: Compound **2a** (55.0 mg, 54 μmol) was dissolved in MeOH (10 mL) at room temperature. After Pd(OH)_2_/C (6.0 mg, 10% wt) was added, the reaction vessel was sealed and changed into a hydrogen atmosphere for the deprotection of both Cbz and Bn groups. Ten hours later, the catalyst was filtered off and the filtrate was concentrated in vacuo. The residue was dissolved in DMF (25 mL) and cooled to 0 °C. After HOAT (7.0 mg, 49 μmol), HATU (93.0 mg, 0.25 mmol), and NMM (32 μL, 0.29 mmol) were sequentially added to the solution, the reaction mixture was stirred at room temperature for 16 h. All volatiles were removed in vacuo, the residue was dissolved in ethyl acetate (100 mL) and washed with saturated aqueous NH_4_Cl (10 mL × 2), water (10 mL × 3) and brine (10 mL). The organic phase was then dried over Na_2_SO_4_ and evaporated in vacuo. The residue was purified by flash chromatography to give the corresponding product **1a** (23.0 mg, 62%). [α]_D_^23^ = −124.4 (*c* 1.0, CHCl_3_). ^1^H NMR (500 MHz, MeOH-*d*_4_): *δ* 7.05 (d, *J* = 8.3 Hz, 2H), 6.70 (d, *J* = 8.4 Hz, 2H), 4.67–4.60 (m, 1H), 4.22–4.09 (m, 1H), 4.02–3.96 (m, 2H), 3.85 (d, *J* = 15.4 Hz, 1H), 3.75–3.70 (m, 1H), 3.65 (s, 3H), 3.41–3.34 (m, 1H), 3.18–3.08 (m, 3H), 2.96–2.91 (m, 1H), 2.36–2.27 (m, 1H), 1.91–1.79 (m, 1H), 1.75–1.52 (m, 7H), 1.42 (s, 9H), 1.35–1.22 (m, 3H), 1.15–1.11 (m, 1H), 0.96–0.88 (m, 15H) ppm; ^13^C NMR (125 MHz, MeOH-*d*_4_) *δ* 174.6, 172.2, 171.3, 169.3, 157.3, 156.0, 130.4, 128.5, 115.1, 84.7, 80.0, 78.6, 62.9, 51.3, 50.7, 50.6, 45.0, 40.7, 38.2, 35.2, 33.3, 31.5, 27.6, 24.9, 24.7, 22.3, 22.0, 20.9, 20.8, 18.3, 12.9 ppm. HRMS (ESI, *m*/*z*) calculated for C_36_H_59_N_4_O_9_^+^ [M+H]^+^: 691.4277 found: 691.4259.

Compound **1b**–**d** was obtained from compound **3** and the corresponding Cbz- protected dipeptide by the same procedures described above.

Compound **1b** was prepared from compound **3** and Cbz-Gly-Gly-OH: [α]_D_^23^ = −10.1 (*c* 0.30, CHCl_3_). ^1^H NMR (400 MHz, MeOH-*d*_4_) *δ* 7.11 (d, *J* = 8.4 Hz, 2H), 6.72 (d, *J* = 6.3 Hz, 2H), 4.60 (t, *J* = 8.4 Hz, 1H), 4.46 (d, *J* = 15.0 Hz, 1H), 4.30–4.20 (m, 2H), 3.79–3.74 (m, 1H), 3.69–3.62 (m, 4H), 3.57 (dd, *J* = 14.5, 9.8 Hz, 2H), 3.22–3.16 (m, 2H), 2.98–2.94 (m, 1H), 2.24–2.08 (m, 1H), 1.90–1.53 (m, 8H), 1.45 (s, 9H), 1.15 (s, 1H), 0.96–0.89 (m, 9H) ppm. ^13^C NMR (125 MHz, MeOH-*d*_4_) *δ* 174.3, 171.2, 169.4, 169.0, 130.5, 128.2, 115.0, 82.5, 80.1, 78.5, 62.2, 51.3, 43.8, 42.6, 41.2, 35.7, 34.1, 33.1, 30.4, 30.3, 27.5, 24.9, 22.2, 21.1, 18.0, 12.9 ppm. HRMS (ESI, *m*/*z*) calculated for C_32_H_51_N_4_O_9_^+^ [M+H]^+^: 635.3651, found: 635.3639.

Compound **1c** was prepared from compound **3** and Cbz-*L*-Ala-Gly-OH: [α]_D_^23^ = −144.3 (*c* 0.34, CHCl_3_). ^1^H NMR (400 MHz, MeOH-*d*_4_): *δ* 7.08 (d, *J* = 8.3 Hz, 2H), 6.72 (d, *J* = 8.3 Hz, 2H), 4.66 (q, *J* = 7.0 Hz, 1H), 4.07–4.00 (m, 1H), 3.94 (q, *J* = 15.5 Hz, 2H), 3.77–3.71 (m, 1H), 3.67 (s, 3H), 3.41–3.35 (m, 1H), 3.20–3.13 (m, 3H), 3.02–2.95 (m, 1H), 2.32 (td, *J* = 13.8, 4.3 Hz, 1H), 1.97–1.85 (m, 1H), 1.78–1.52 (m, 5H), 1.44 (s, 9H), 1.36–1.27 (m, 4H), 1.20–1.12 (m, 1H), 0.96–0.90 (m, 9H) ppm; ^13^C NMR (100 MHz, MeOH-*d*_4_): *δ* 174.3, 172.2, 171.1, 169.2, 157.1, 155.9, 130.3, 128.3, 114.8, 84.5, 79.9, 78.4, 62.6, 51.1, 50.5, 44.9, 40.5, 35.0, 33.2, 31.3, 27.4, 24.8, 22.2, 20.8, 18.2, 14.1, 12.8 ppm. HRMS (ESI, *m*/*z*) calculated for C_33_H_53_N_4_O_9_^+^ [M+H]^+^: 649.3807, found: 649.3794.

Compound **1d** was prepared from compound **3** and Cbz-Gly-*L*-Ala-OH: [α]_D_^23^ = −84.0 (*c* 0.25, MeOH). ^1^H NMR (500 MHz, MeOH-*d*_4_) *δ* 7.12 (d, *J* = 8.4 Hz, 2H), 6.69 (d, *J* = 8.4 Hz, 2H), 4.56 (q, *J* = 7.5 Hz, 1H), 4.13–4.07 (m, 1H), 3.90 (dd, *J* = 53.8, 15.3 Hz, 2H), 3.65– 3.56 (m, 4H), 3.42–3.36 (m, 1H), 3.20–3.13 (m, 3H), 3.02–2.97 (m, 1H), 2.26 (td, *J* = 14.0, 4.0 Hz, 1H), 1.88 (td, *J* = 14.4, 3.9 Hz, 1H), 1.73 (dt, *J* = 12.6, 6.5 Hz, 1H), 1.66–1.48 (m, 5H), 1.43 (s, 9H), 1.39 (d, *J* = 7.6 Hz, 3H), 1.35–1.28 (m, 1H), 1.17–1.11 (m, 1H), 0.95–0.90 (m, 9H) ppm. ^13^C NMR (125 MHz, MeOH-*d*_4_) *δ* 172.0, 171.2, 169.8, 157.2, 156.1, 130.8, 128.2, 114.8, 83.4, 81.1, 78.5, 62.6, 52.5, 51.2, 50.3, 43.2, 41.0, 34.8, 33.3, 30.0, 27.6, 24.9, 22.3, 21.0, 18.2, 16.8, 12.8 ppm. HRMS (ESI, *m*/*z*) calculated for C_33_H_53_N_4_O_9_^+^ [M+H]^+^: 649.3807, found: 649.3789.

Methyl (*S*)-2-((2*S*,5*S*,13*S*)-13-((*S*)-1-amino-3-methylbutyl)-5-isobutyl-3,6,9-trioxo-2-propyl-1-oxa-4,7,10-triazacyclotridecan-10-yl)-3-(4-hydroxyphenyl)propanoate **18**: Compound **1a** (9.0 mg, 0.13 mmol) was dissolved in DCM (2 mL) at 0 °C. After TFA (0.2 mL) was dropwise added, the reaction mixture was stirred for 2 h and then concentrated in vacuo. The residue was purified by preparative HPLC to afford the desired cyclic peptide **18** (6.0 mg, 78%). The preparative HPLC was performed on an Agilent 1200 system equipped with a reverse-phase Agilent SB-C18 column (21.2 × 250 mm), and it was eluted with H_2_O-MeOH (contains 0.1% of TFA) (42:58) at a flow rate of 10 mL/min. [α]_D_^23^ = −123 (*c* 0.10, MeOH). ^1^H NMR (400 MHz, MeOH-*d*_4_) *δ* 7.01 (d, *J* = 8.4 Hz, 2H), 6.74 (d, *J* = 8.5 Hz, 2H), 4.67–4.57 (m, 1H), 3.99 (q, *J* = 15.5 Hz, 2H), 3.87–3.75 (m, 2H), 3.71 (s, 3H), 3.25–3.17 (m, 3H), 2.93–2.84 (m, 1H), 2.27–2.15 (m, 1H), 1.88–1.59 (m, 7H), 1.50–1.30 (m, 4H), 1.00–0.95 (m, 9H), 0.94–0.88 (m, 6H) ppm. ^13^C NMR (100 MHz, MeOH-*d*_4_) *δ* 173.2, 172.4, 170.9, 169.5, 156.1, 130.1, 128.6, 115.1, 84.9, 75.7, 63.6, 51.9, 51.3, 50.5, 46.3, 44.8, 38.9, 37.7, 34.6, 33.1, 30.8, 24.5, 23.9, 21.9, 21.2, 21.1, 20.5, 17.9, 12.7 ppm. HRMS (ESI, *m*/*z*) calculated for C_31_H_51_N_4_O_7_^+^ [M+H]^+^: 591.3752, found: 591.3757.

### 3.3. Two-Dimensional COSY and NOESY Analysis of Compound 15

The COSY and NOESY experiments were recorded in acetone-*d*_6_/methanol-*d*_4_ (2:1). The co-solvent system was used here to distinguish the signals of H_a_ from H_2″_, H_c_ from H_1″_ (which were found severely overlapped in either CDCl_3_ or Acetone-*d*_6_). From the ^1^H NMR and COSY spectra, H_a_ was assigned to 3.80 ppm (1H, dt, *J*_1_ = 10 Hz, *J*_2_ = 5.2 Hz), H_c_ were assigned to 1.69–1.71 ppm (2H, m) and H_b_ was assigned to 4.03 ppm (1H, dd, *J*_1_ = 8.0 Hz, *J*_2_ = 4.4 Hz). The NOESY spectrum showed clear correlations of H_a_-H_c_ and H_c_-H_b_, no direct correlation between H_a_ and H_b_, and these correlation signals unambiguously proved that H_b_ is at the equatorial position (six-membered ring’s chair-confirmation), and thus the stereochemistry at C-2 was determined as 2*S*.

**Figure:**
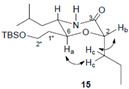


## 4. Conclusions

The development of new methods for the helical stabilization of a linear peptide could provide additional opportunities for the discovery of peptide-based therapeutics targeting PPI. Design and synthesis of hydrogen-bond surrogates (HBSs) are one of the most successful approaches. In this paper, we developed a new helix template based on the replacement of the weak (*i*, *i* + 4) H-bond with a covalent carbon–carbon linkage; and also employed a covalent ether bond to mimic one amide-bond. In comparing the precedent hydrogen-bond surrogates in which the hydrogen bond was replaced with hydra-zine, carbon–carbon links, and thioether linkage, our new helix template could be placed in a position other than the *N*-terminus of a short peptide. The CD studies show that the helix template adopts stable conformations in aqueous buffer at exceptionally high temperatures. We also found that the stereogenic centers presented in the macrocycle are vital for the stability of helical conformation. The scaffold obtained has been proven as a single-turn helical mimetic. The further coupling of additional short peptide fragments and beta-strand mimetic aiming to develop inhibitors of p53 degradation based on the PIP motif of PCNA interaction proteins will be reported in due course.

## Data Availability

Not applicable.

## Supplementary Materials

The following supporting information can be downloaded at: https://www.mdpi.com/article/10.3390/molecules28020780/s1, Copies of NMR spectra (^1^H and ^13^C) of **8**–**10**, **6**, **14**, **15**, **5**, **16**, **3**, **2a**, **1a**, **1b**, **1c, 1d**, **18**.

## Sample Availability

Samples of the compounds are not available from the authors.
