# Supplementary material for: Design and Synthesis of Novel Helix Mimetics Based on the Covalent H-Bond Replacement and Amide Surrogate
Source: Molecules. 2023 Jan 12;28(2):780. doi: 10.3390/molecules28020780 (PMC9863496; doi:10.3390/molecules28020780)

Avance 500, Bruker
allytrbutyltin, solvent:CDCl3

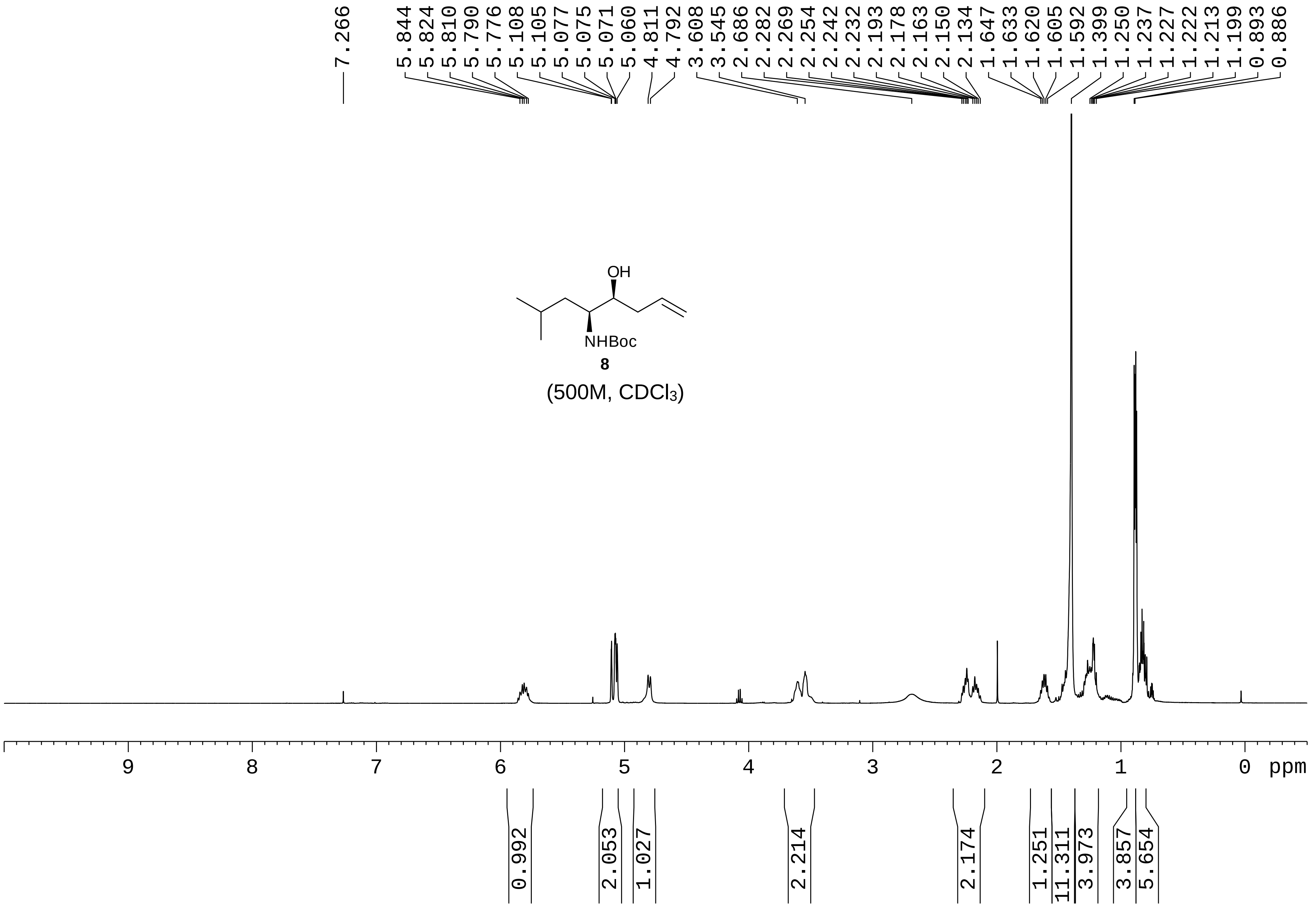

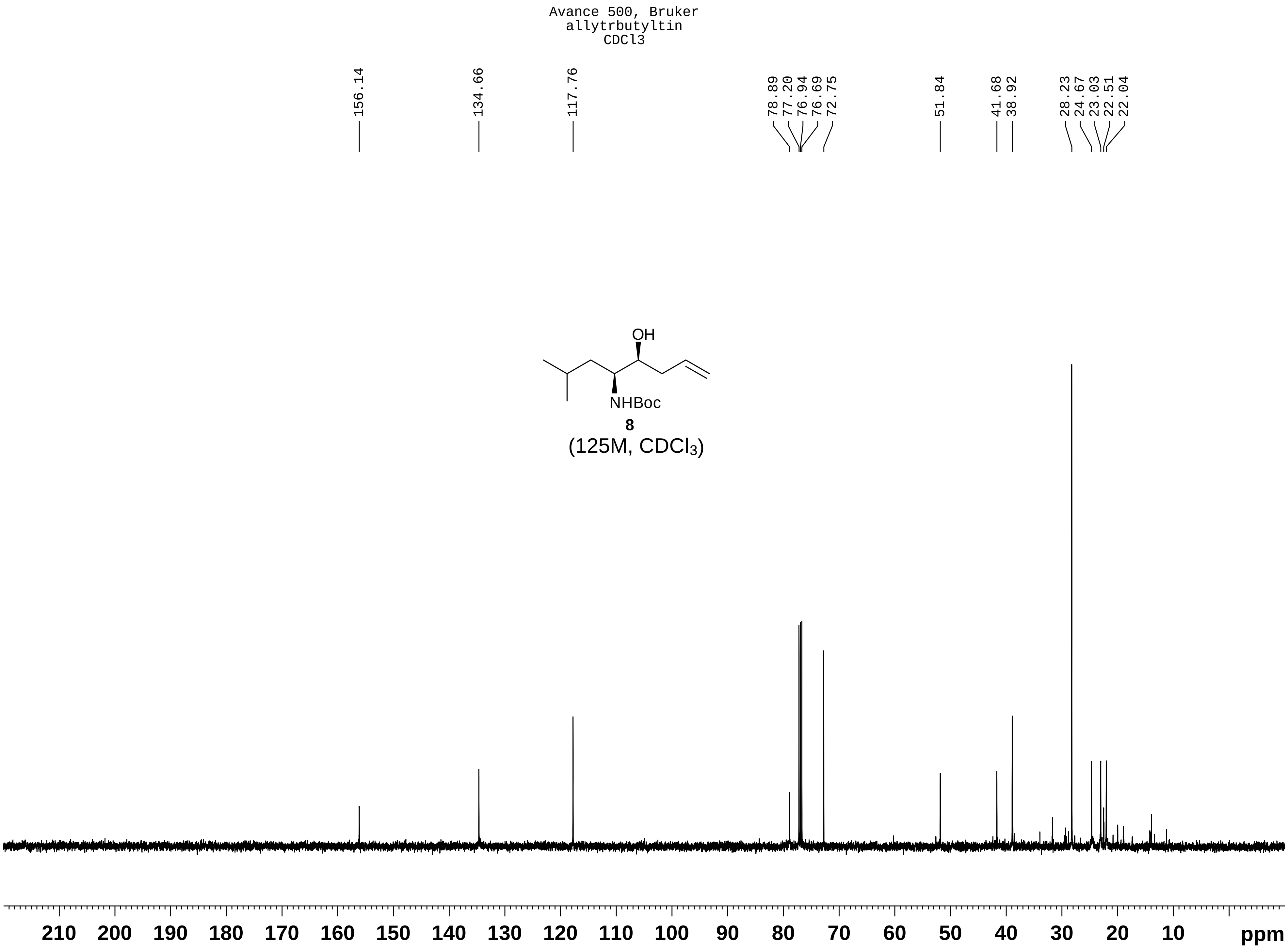
Avance 500, Bruker
allytrbutyltin
CDCl3
156.14
134.66
117.76
78.89
77.20
76.94
76.69
72.75
51.84
41.68
38.92
28.23
24.67
23.03
22.51
22.04
OH
NHBoc
8
(125M, CDCl3)
210 200 190 180 170 160 150 140 130 120 110 100 90 80 70 60 50 40 30 20 10
ppm

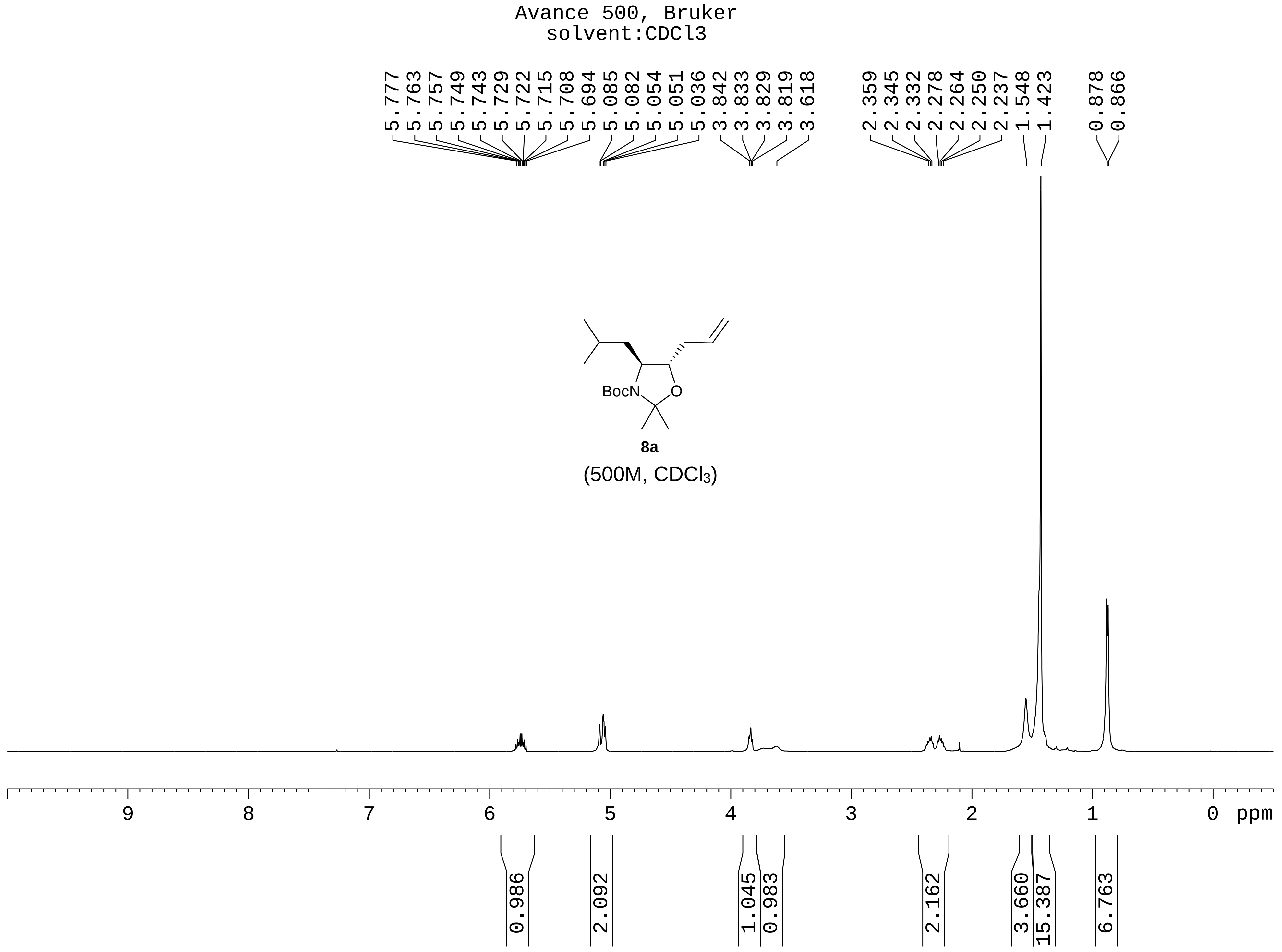
Avance 500, Bruker
solvent:CDCl3
5.777 5.763 5.757 5.749 5.743 5.729 5.722 5.715 5.708 5.694 5.085 5.082 5.054 5.051 5.036 3.842 3.833 3.829 3.819 3.618 2.359 2.345 2.332 2.278 2.264 2.250 2.237 1.548 1.423 0.878 0.866
BocN
O
8a
(500M, CDCl3)
9 8 7 6 5 4 3 2 1 0 ppm
0.986 2.092 1.045 0.983 2.162 3.660 15.387 6.763

Avance 500, Bruker
solvent:CDCl3

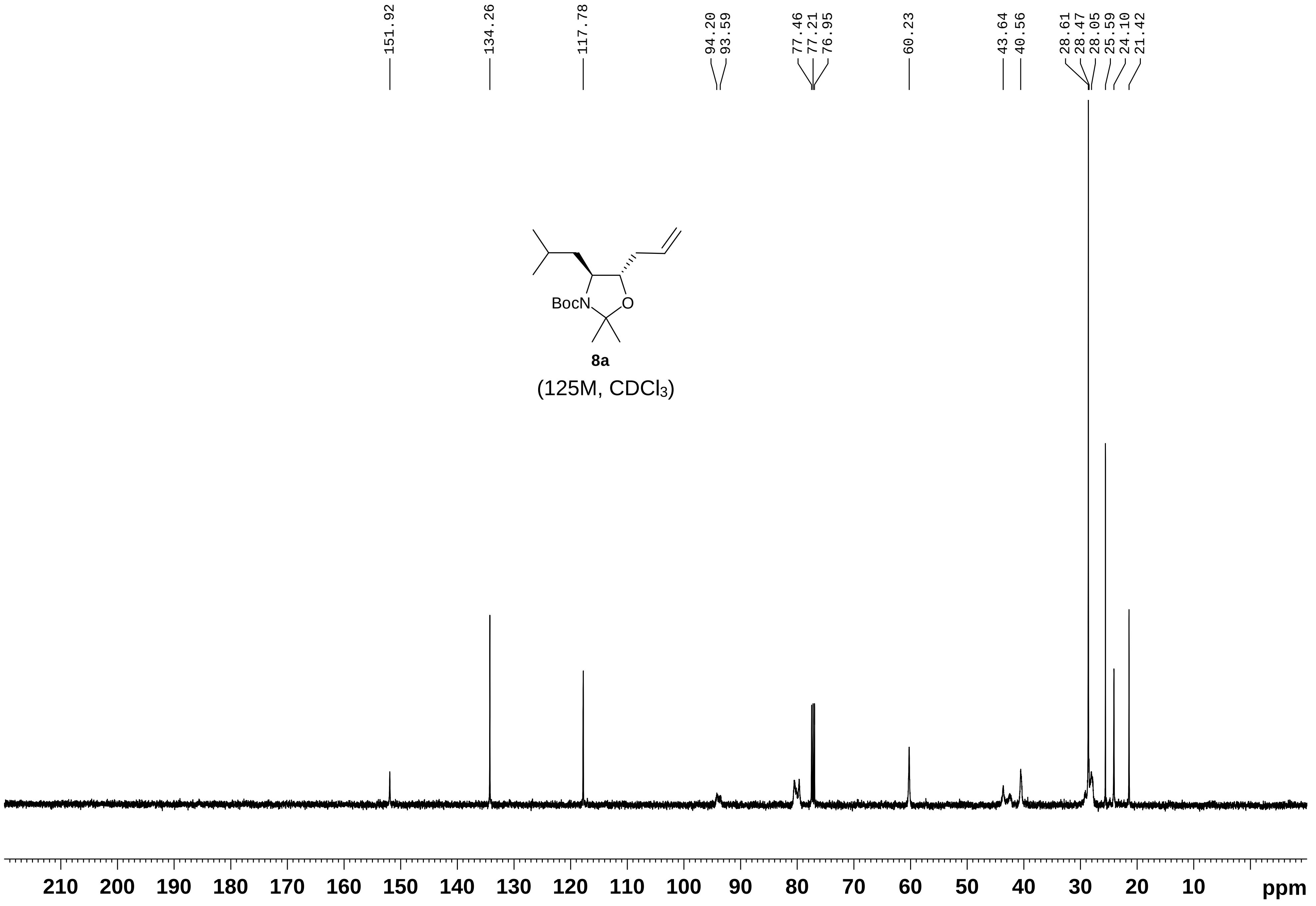

Avance 500, Bruker
solvent:CDCl3

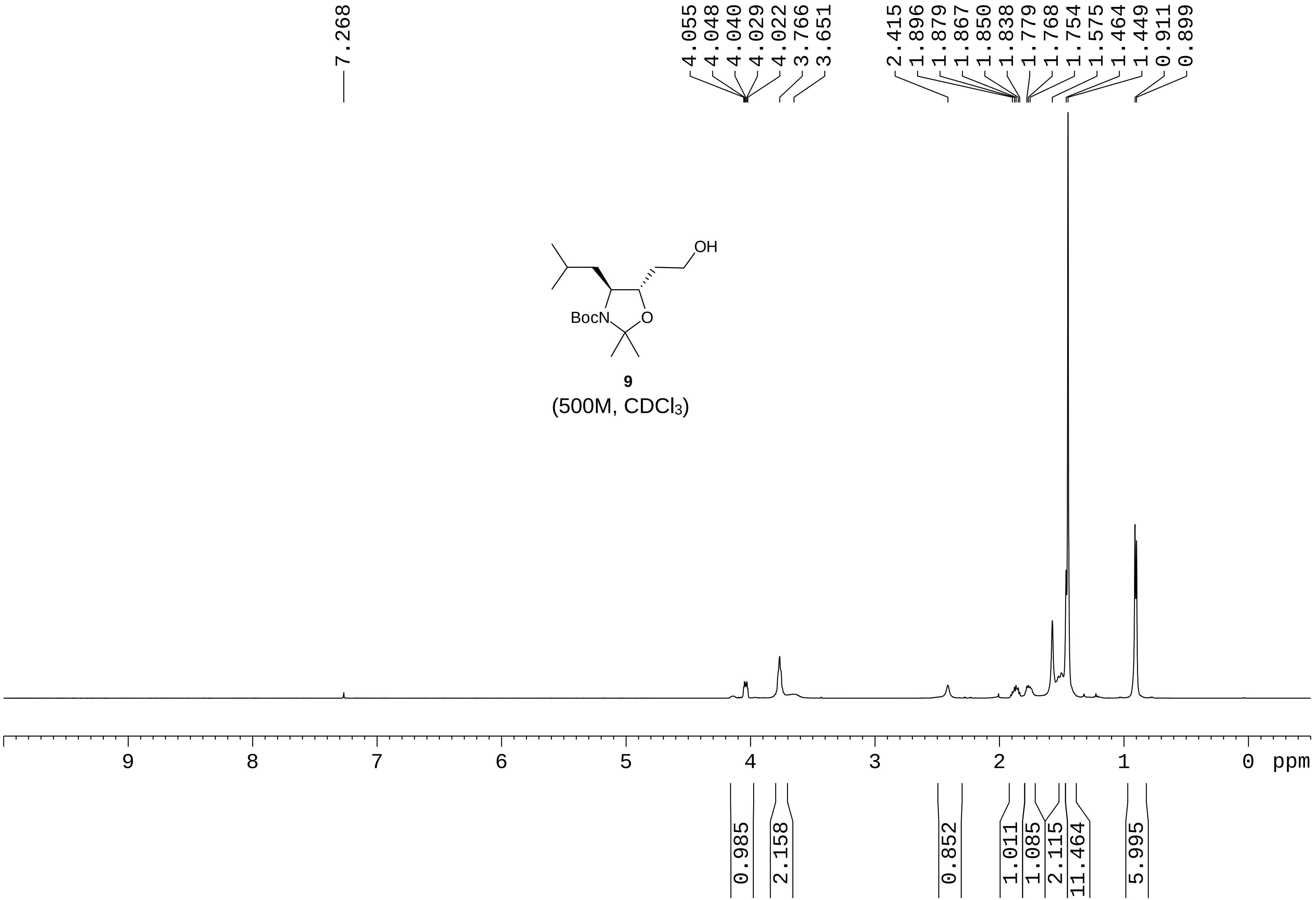

9
(500M, $CDCl_3$)

Avance 500, Bruker
CDCl3

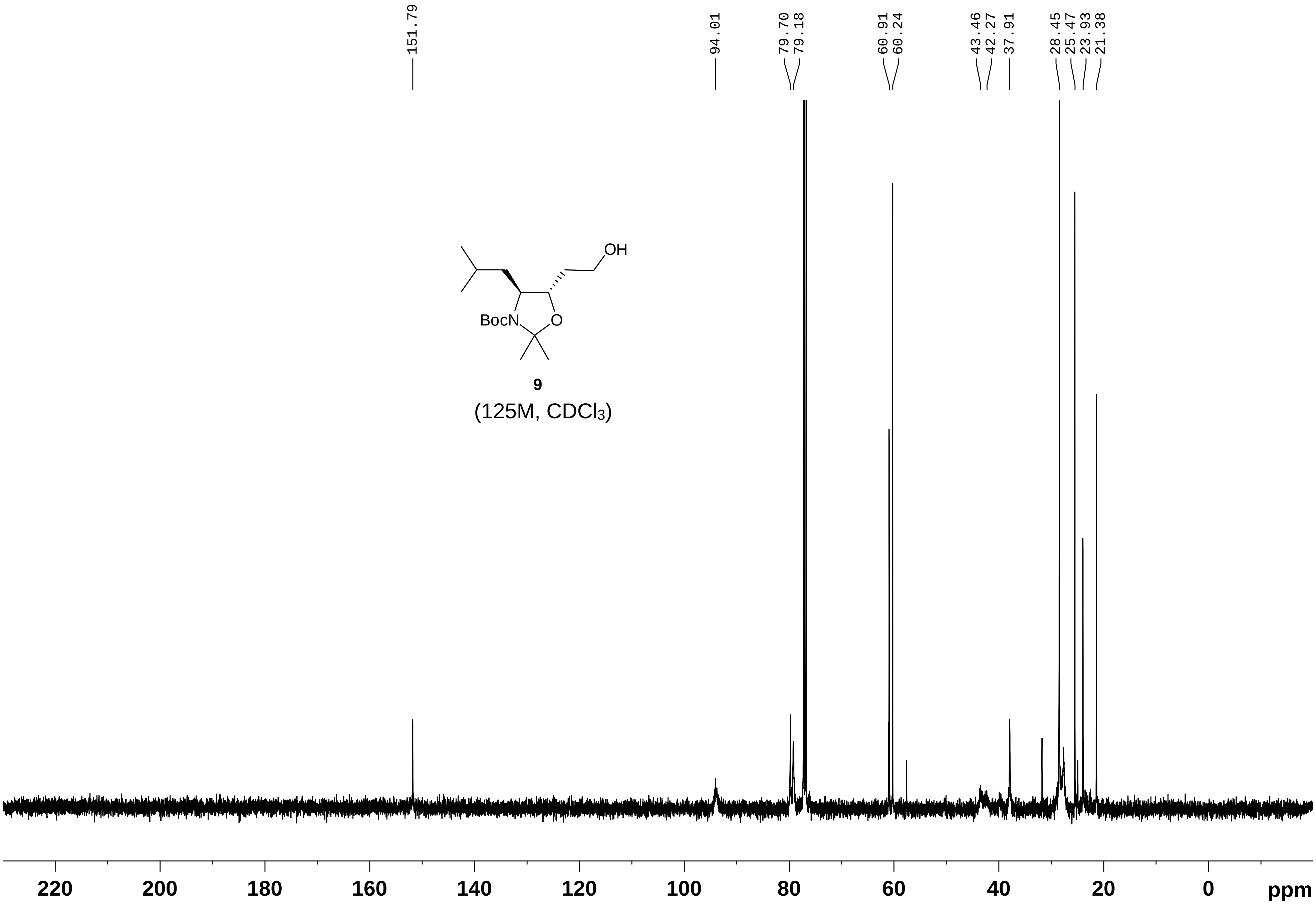

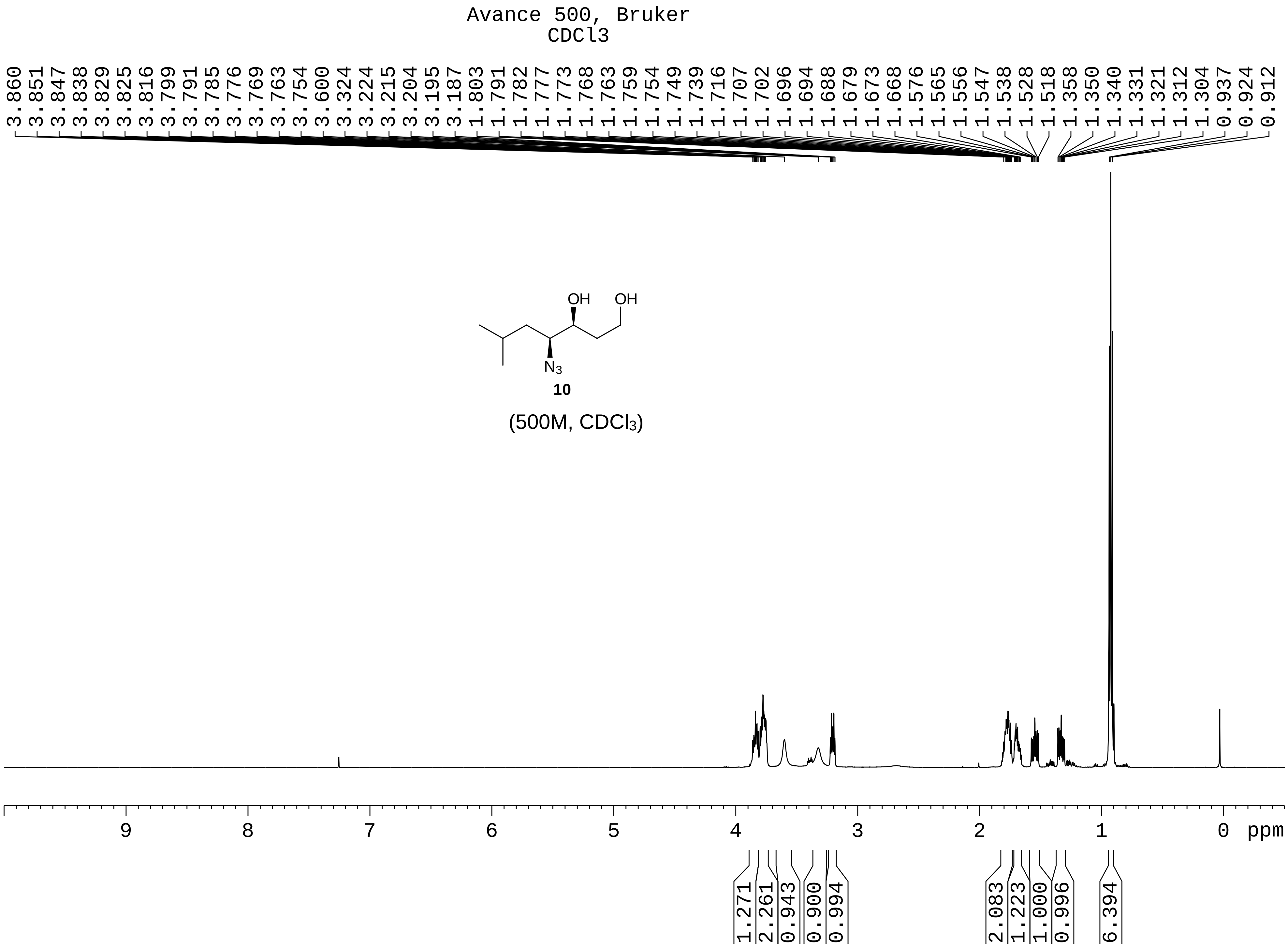

Avance 500, Bruker
CDCl3
3.860 3.851 3.847 3.838 3.829 3.825 3.816 3.799 3.791 3.785 3.776 3.769 3.763 3.754 3.600 3.324 3.224 3.215 3.204 3.195 3.187 1.803 1.791 1.782 1.777 1.773 1.768 1.763 1.759 1.754 1.749 1.739 1.716 1.707 1.702 1.696 1.694 1.688 1.679 1.673 1.668 1.576 1.565 1.556 1.547 1.538 1.528 1.518 1.358 1.350 1.340 1.331 1.321 1.312 1.304 0.937 0.924 0.912
OH
OH
N3
10
(500M, CDCl3)
9 8 7 6 5 4 3 2 1 0 ppm
1.271 2.261 0.943 0.900 0.994 2.083 1.223 1.000 0.996 6.394

Avance 500, Bruker
CDCl3

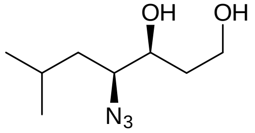

10

(125M, $CDCl_3$)

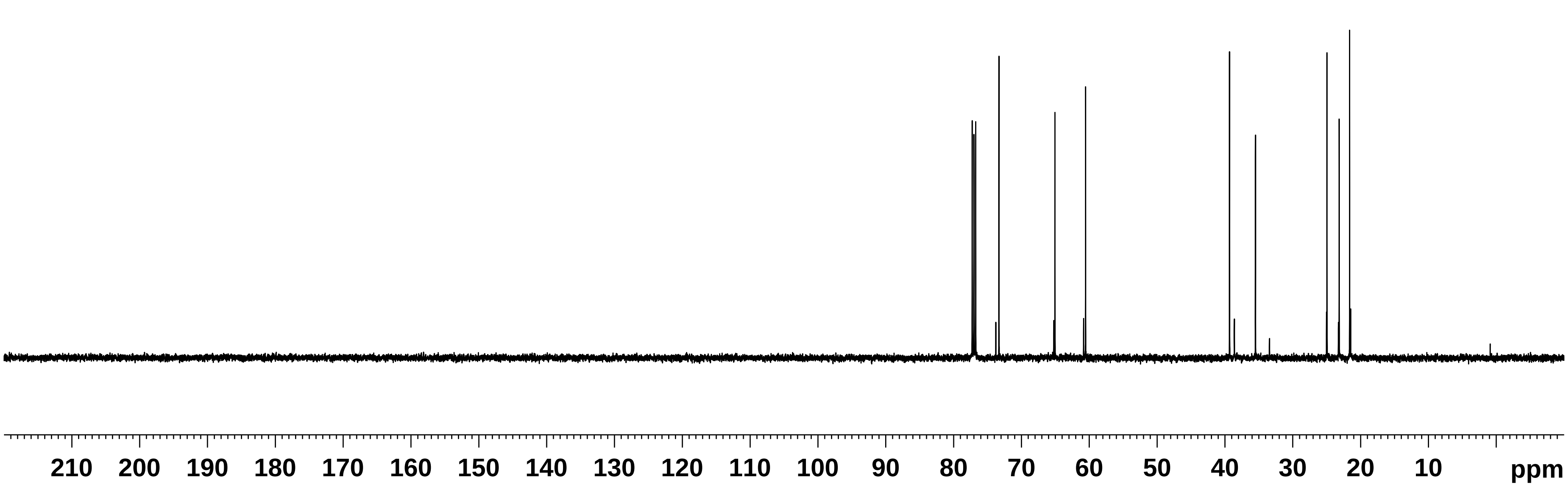

Avance 500, Bruker
CDCl3

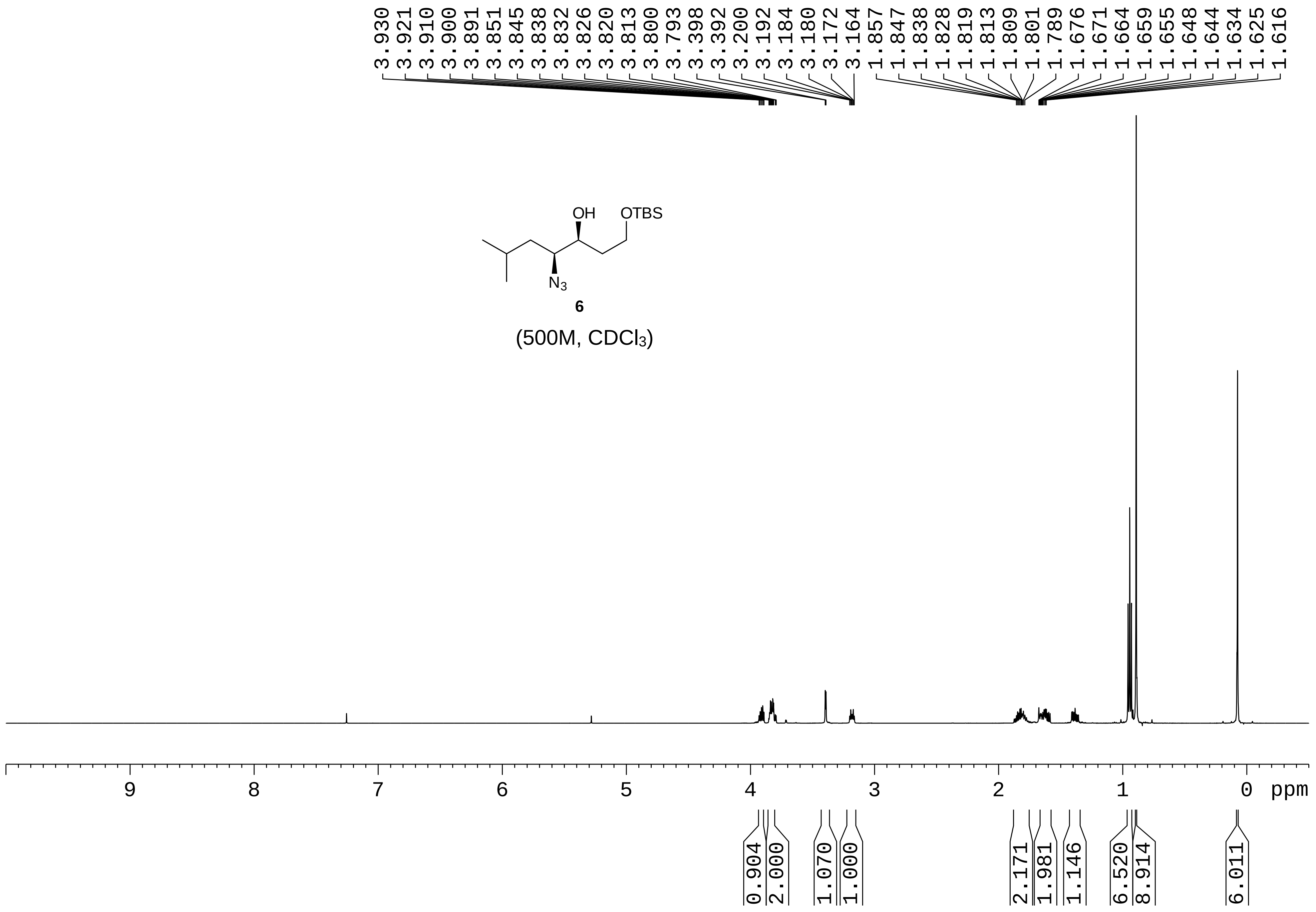

Avance 500, Bruker
CDCl3

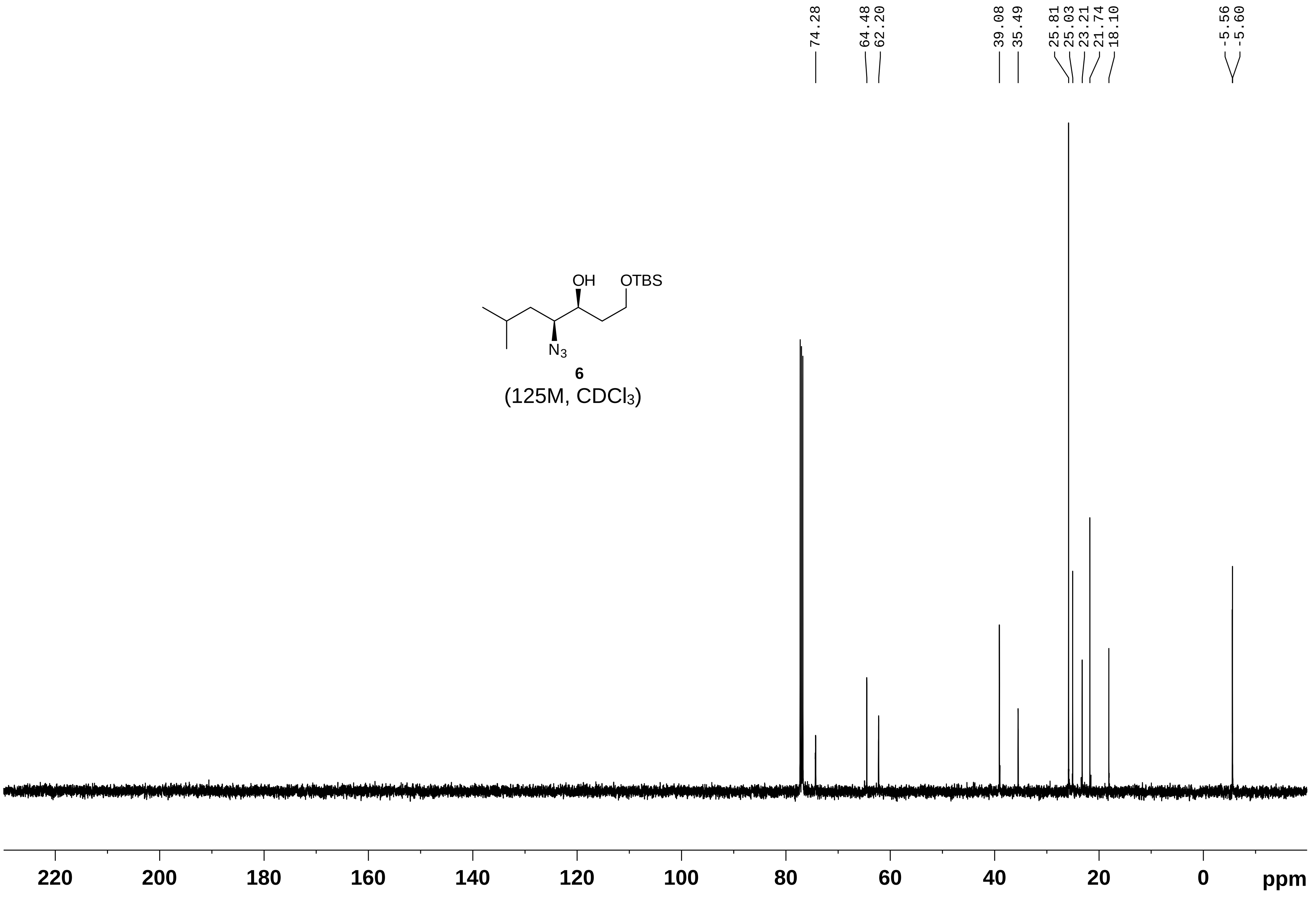

Avance 500, Bruker
CDCl3

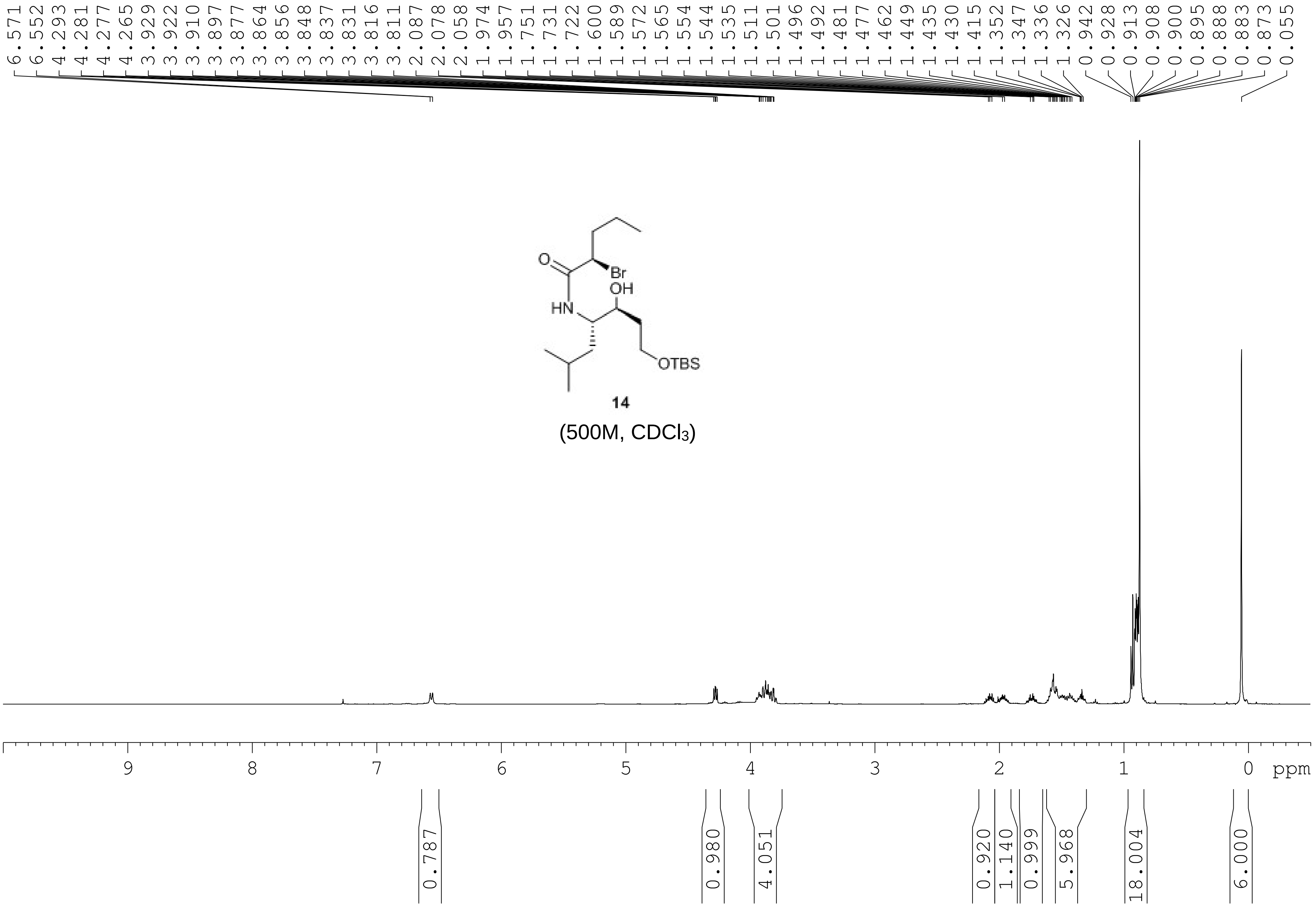

Avance 500, Bruker
CDCl3

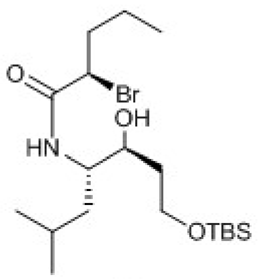

14

(125M, $CDCl_3$)

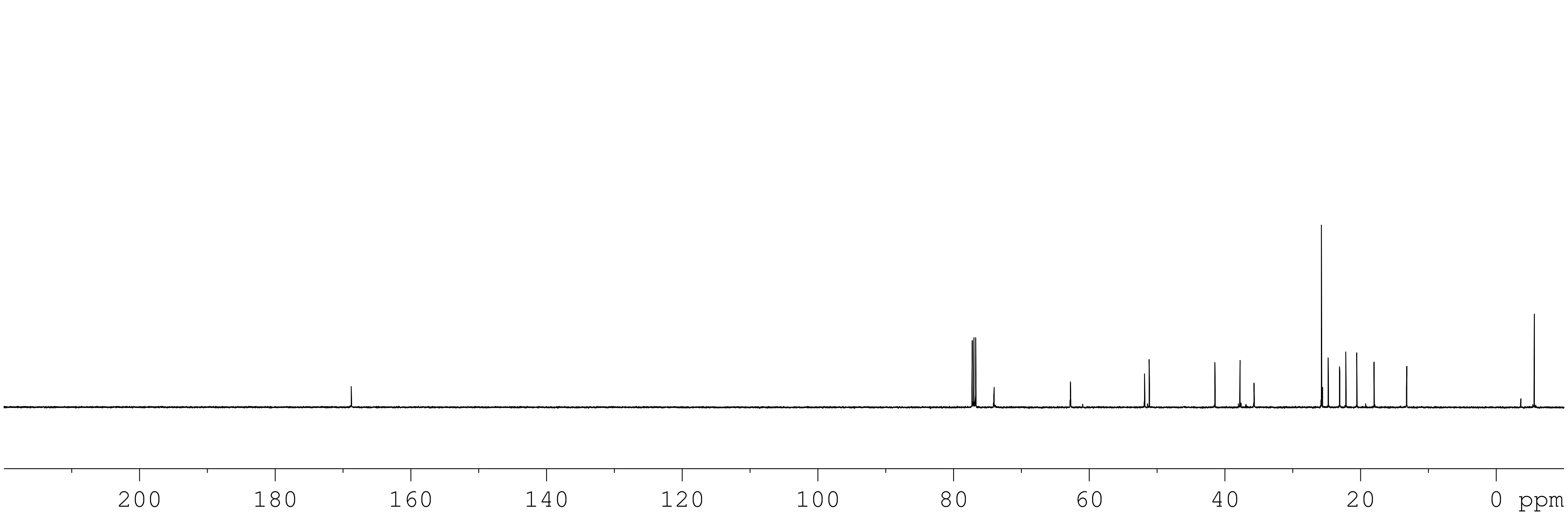

Avance 500, Bruker
CDCl3

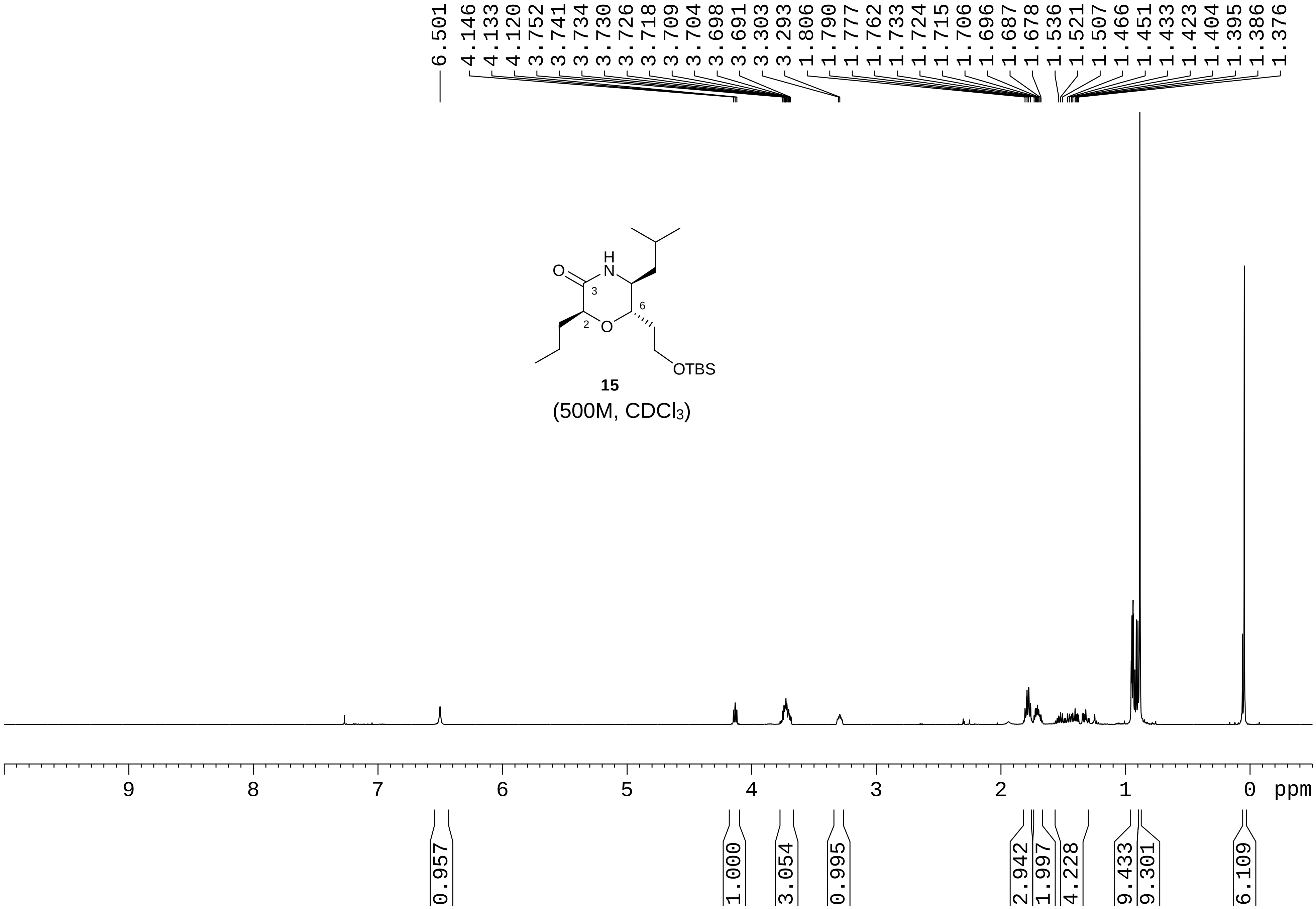

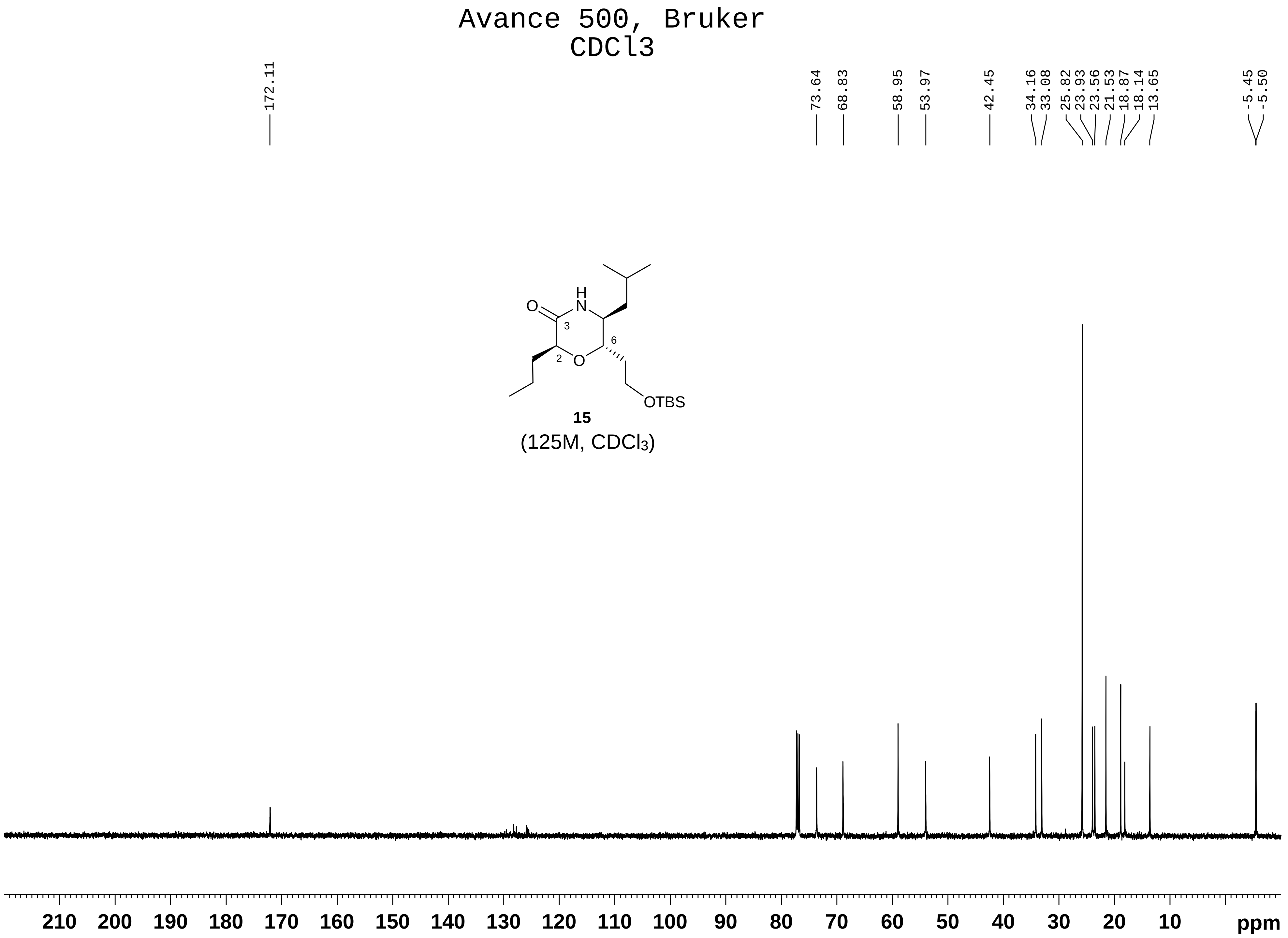

Avance 500, Bruker
CDCl3
172.11
73.64
68.83
58.95
53.97
42.45
34.16
33.08
25.82
23.93
23.56
21.53
18.87
18.14
13.65
-5.45
-5.50
H
N
O
3
6
2
O
OTBS
15
(125M, CDCl3)
210 200 190 180 170 160 150 140 130 120 110 100 90 80 70 60 50 40 30 20 10
ppm

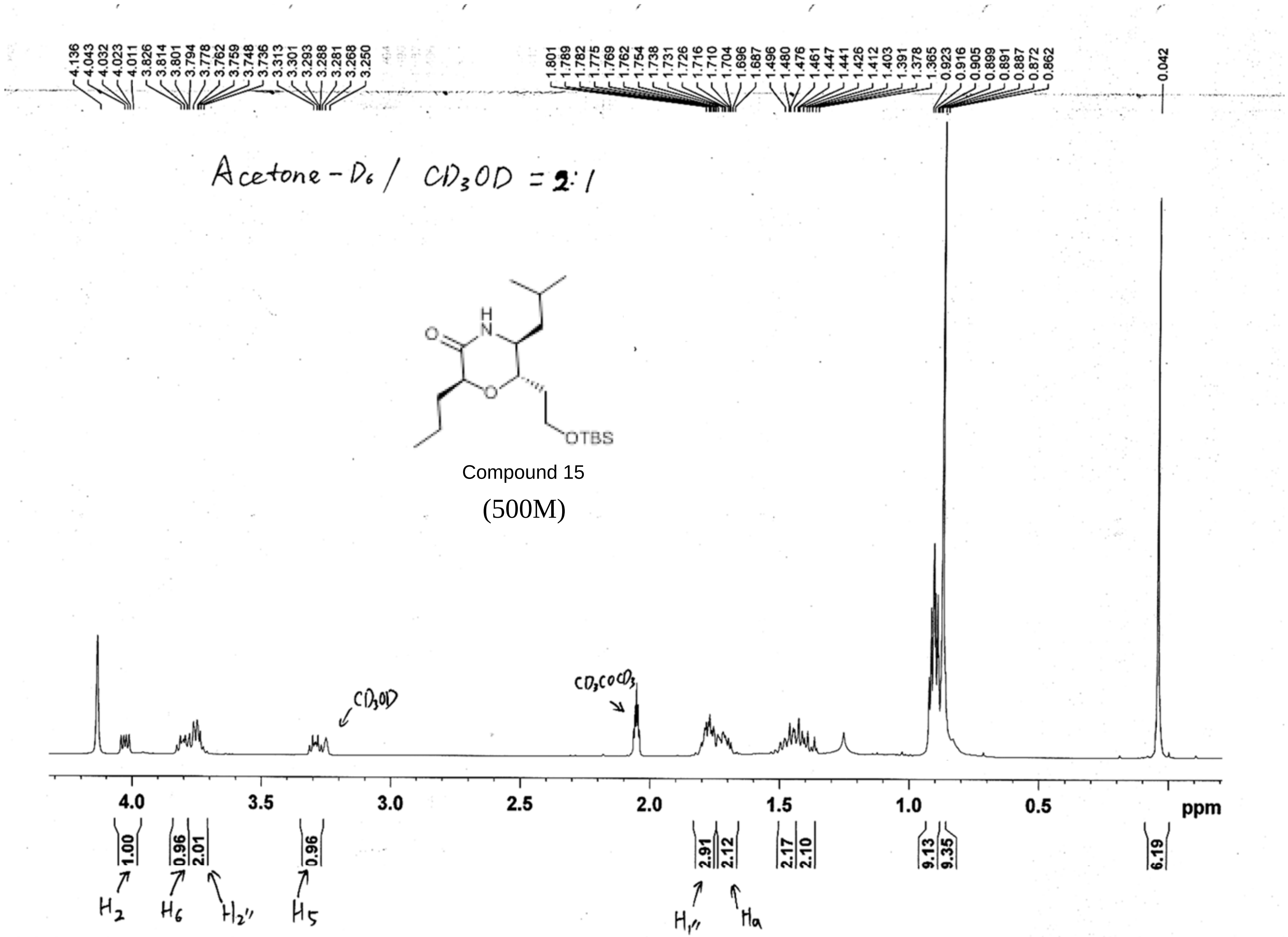

4.136 4.043 4.032 4.023 4.011 3.826 3.814 3.801 3.794 3.778 3.762 3.759 3.748 3.736 3.313 3.301 3.293 3.288 3.281 3.268 3.250
1.801 1.789 1.782 1.775 1.769 1.762 1.754 1.738 1.731 1.726 1.716 1.710 1.704 1.696 1.687 1.496 1.480 1.476 1.461 1.447 1.441 1.426 1.412 1.403 1.391 1.378 1.365 0.923 0.916 0.905 0.899 0.891 0.887 0.872 0.862
0.042
Acetone-D6 / CD3OD = 2:1
O
H
N
O
OTBS
Compound 15
(500M)
CD3OD
CD3COCD3
4.0
3.5
3.0
2.5
2.0
1.5
1.0
0.5
ppm
1.00
0.96
2.01
0.96
2.91
2.12
2.17
2.10
9.13
9.35
6.19
H2
H6
H2''
H5
H1''
Ha

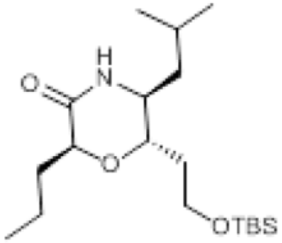

Compound 15

(500M)

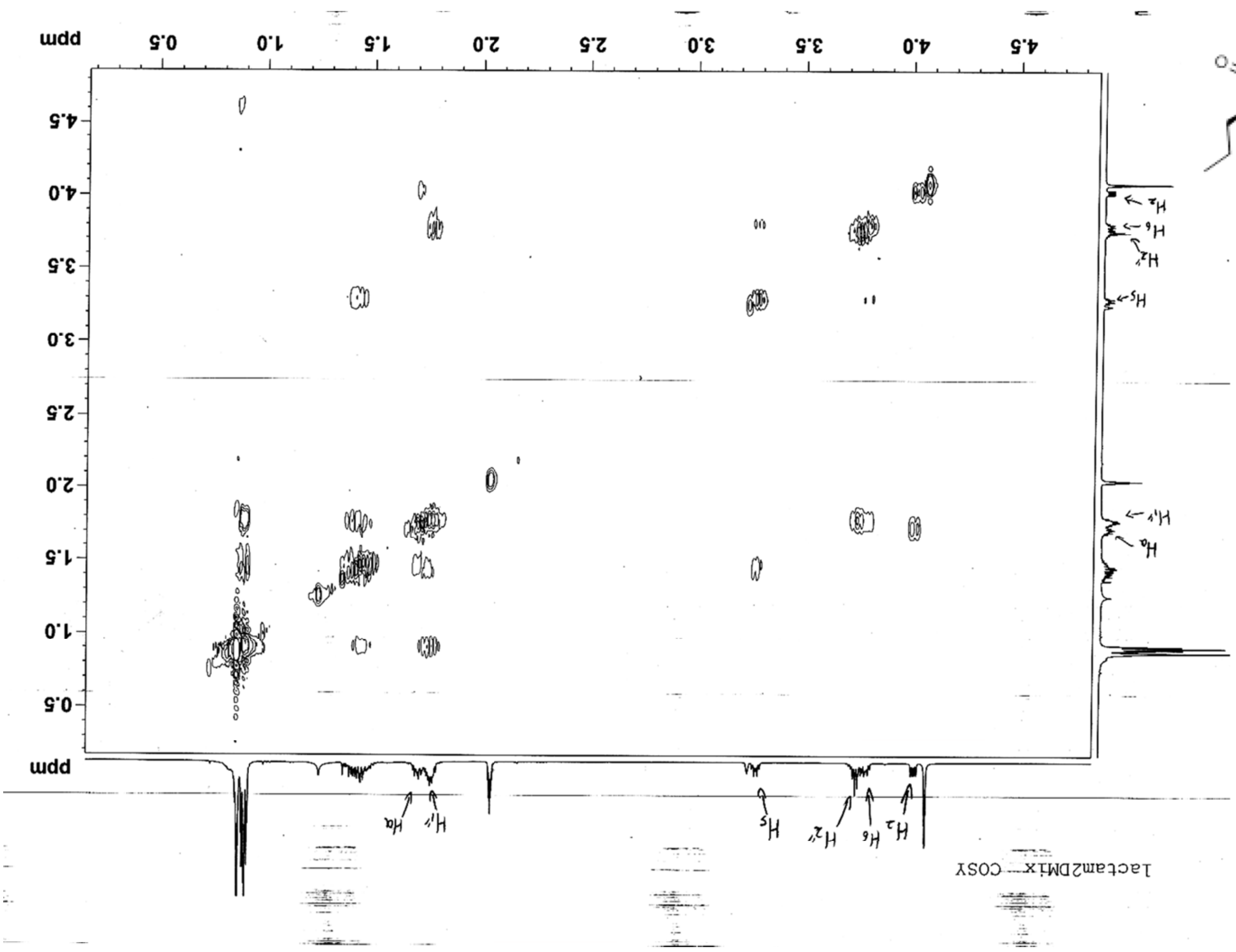

2D COSY

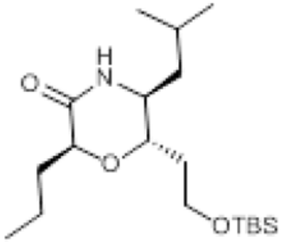

Compound 15

(500M)

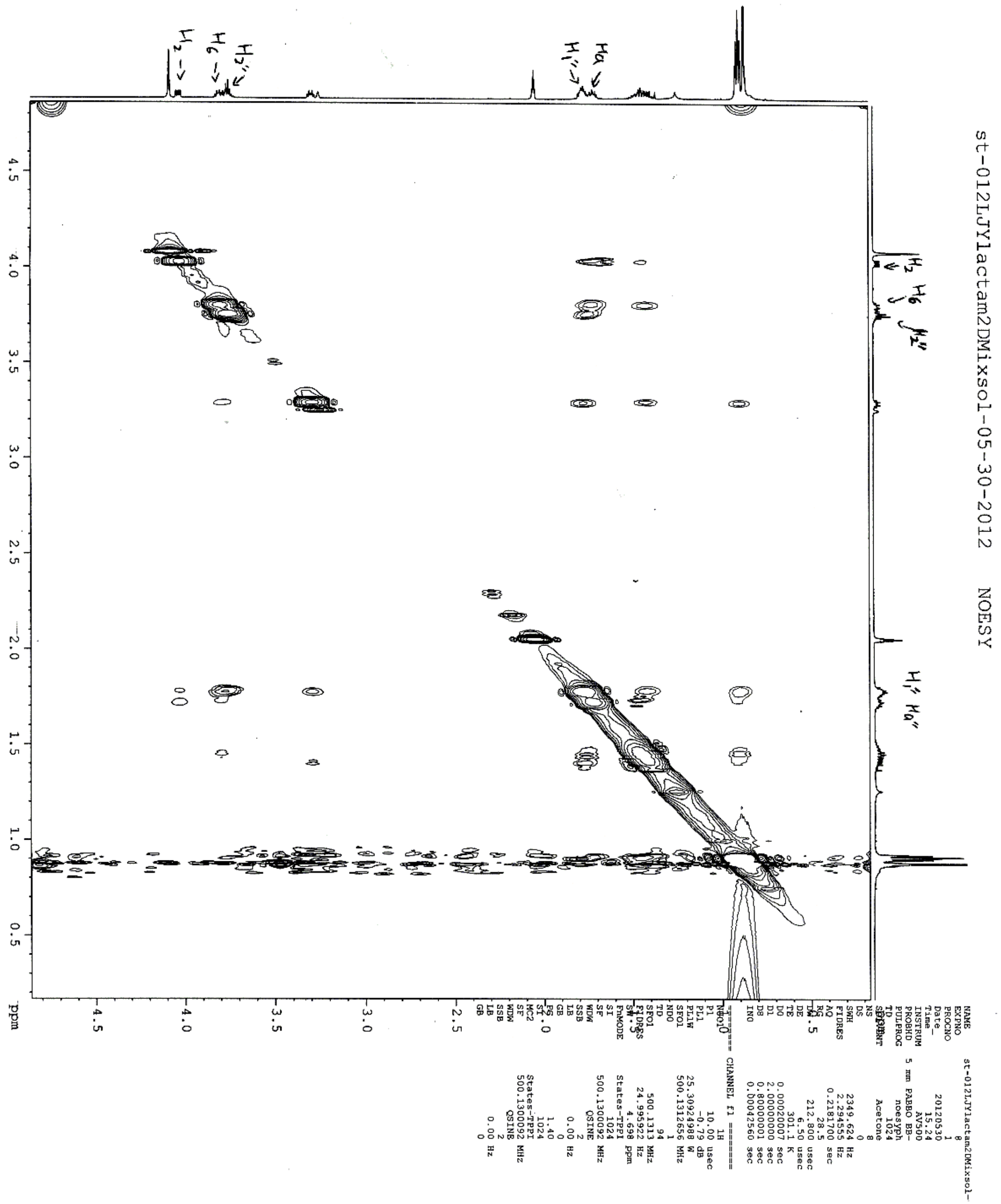

2D NOESY

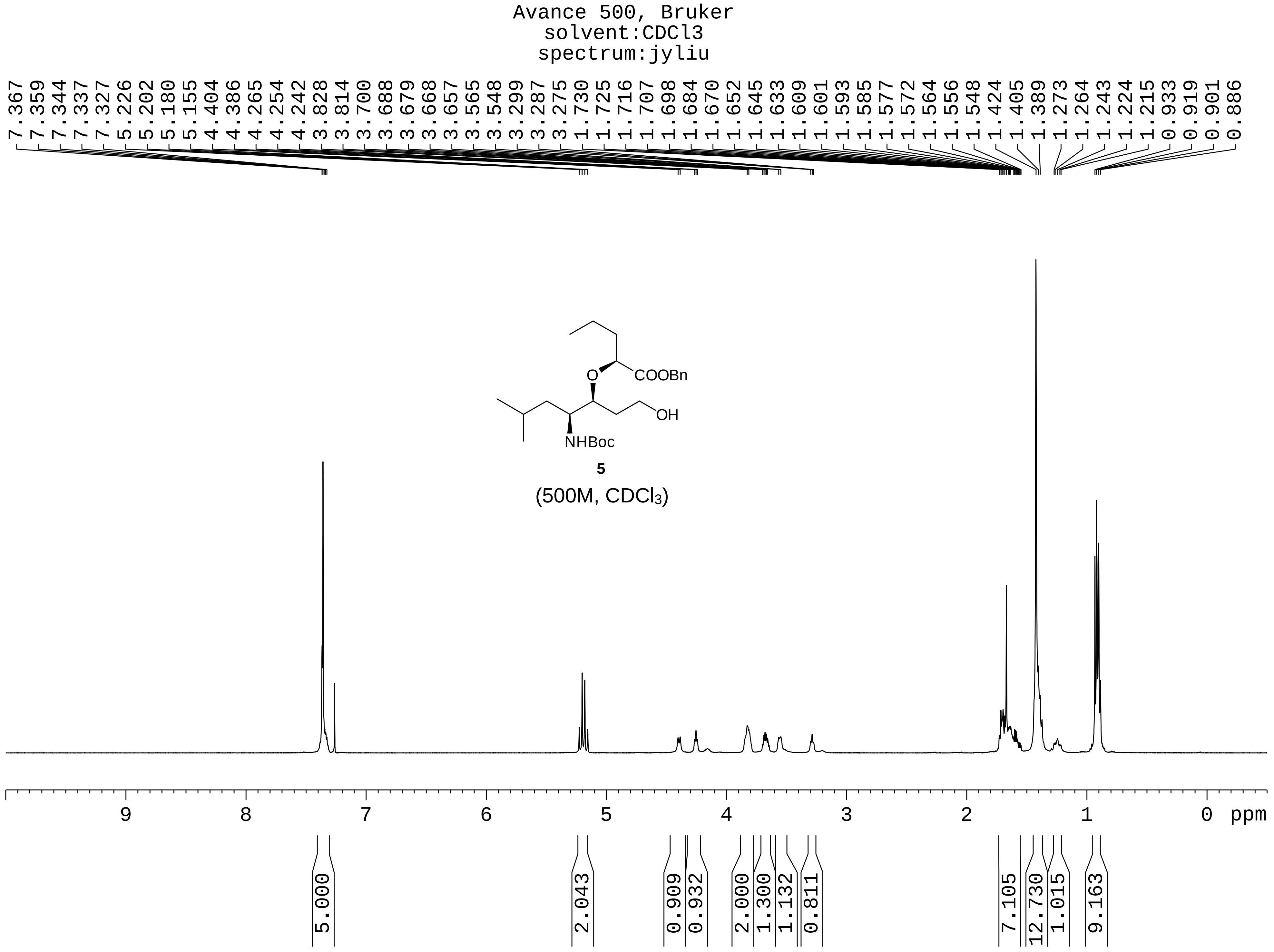
Avance 500, Bruker
solvent:CDCl3
spectrum:jyliu
7.367 7.359 7.344 7.337 7.327 5.226 5.202 5.180 5.155 4.404 4.386 4.265 4.254 4.242 3.828 3.814 3.700 3.688 3.679 3.668 3.657 3.565 3.548 3.299 3.287 3.275 1.730 1.725 1.716 1.707 1.698 1.684 1.670 1.652 1.645 1.633 1.609 1.601 1.593 1.585 1.577 1.572 1.564 1.556 1.548 1.424 1.405 1.389 1.273 1.264 1.243 1.224 1.215 0.933 0.919 0.901 0.886
O
COOBn
OH
NHBoc
5
(500M, CDCl3)
9 8 7 6 5 4 3 2 1 0 ppm
5.000 2.043 0.909 0.932 2.000 1.300 1.132 0.811 7.105 12.730 1.015 9.163

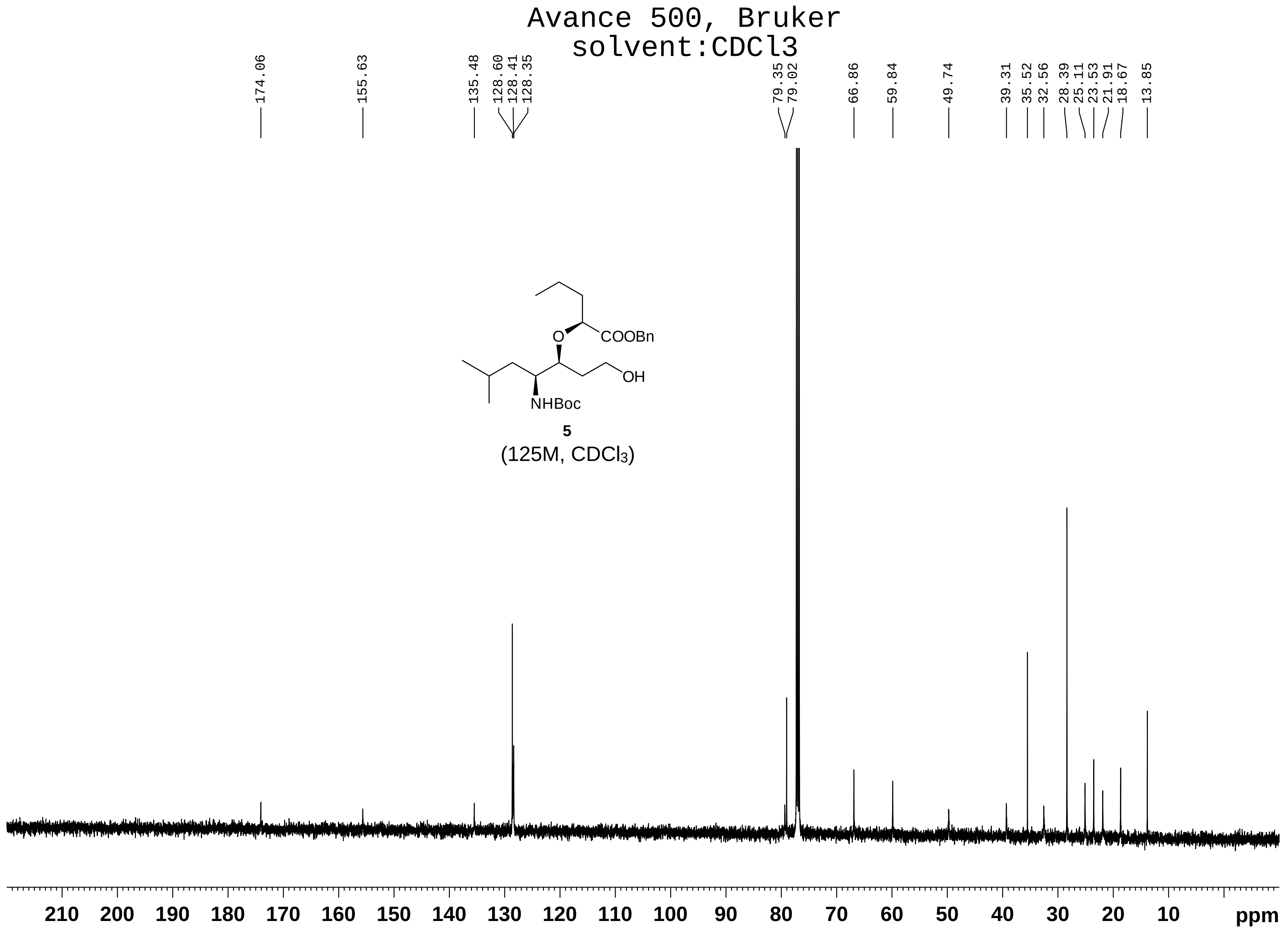

Avance 500, Bruker
solvent:CDCl3
174.06
155.63
135.48
128.60
128.41
128.35
79.35
79.02
66.86
59.84
49.74
39.31
35.52
32.56
28.39
25.11
23.53
21.91
18.67
13.85
COOBn
O
OH
NHBoc
5
(125M, CDCl3)
210
200
190
180
170
160
150
140
130
120
110
100
90
80
70
60
50
40
30
20
10
ppm

SZPKU Bruker 400
Solvent: CDCl3
Spectrum: jyliu

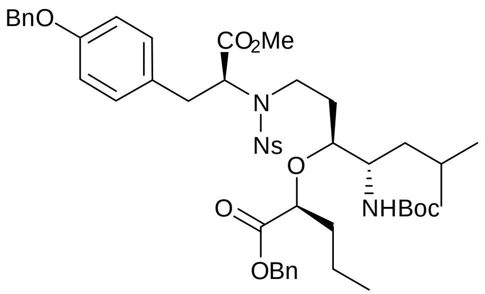

16

(400M, $CDCl_3$)

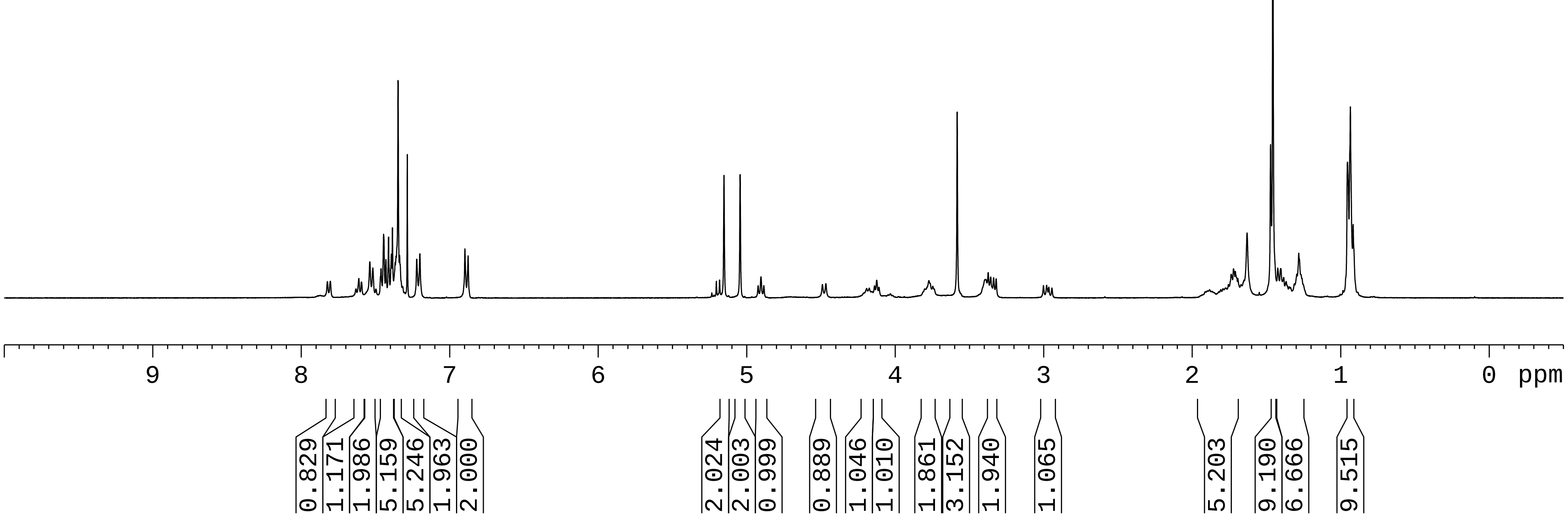

SZPKU Bruker 400
Solvent CDCl3
Spectrum: jyliu

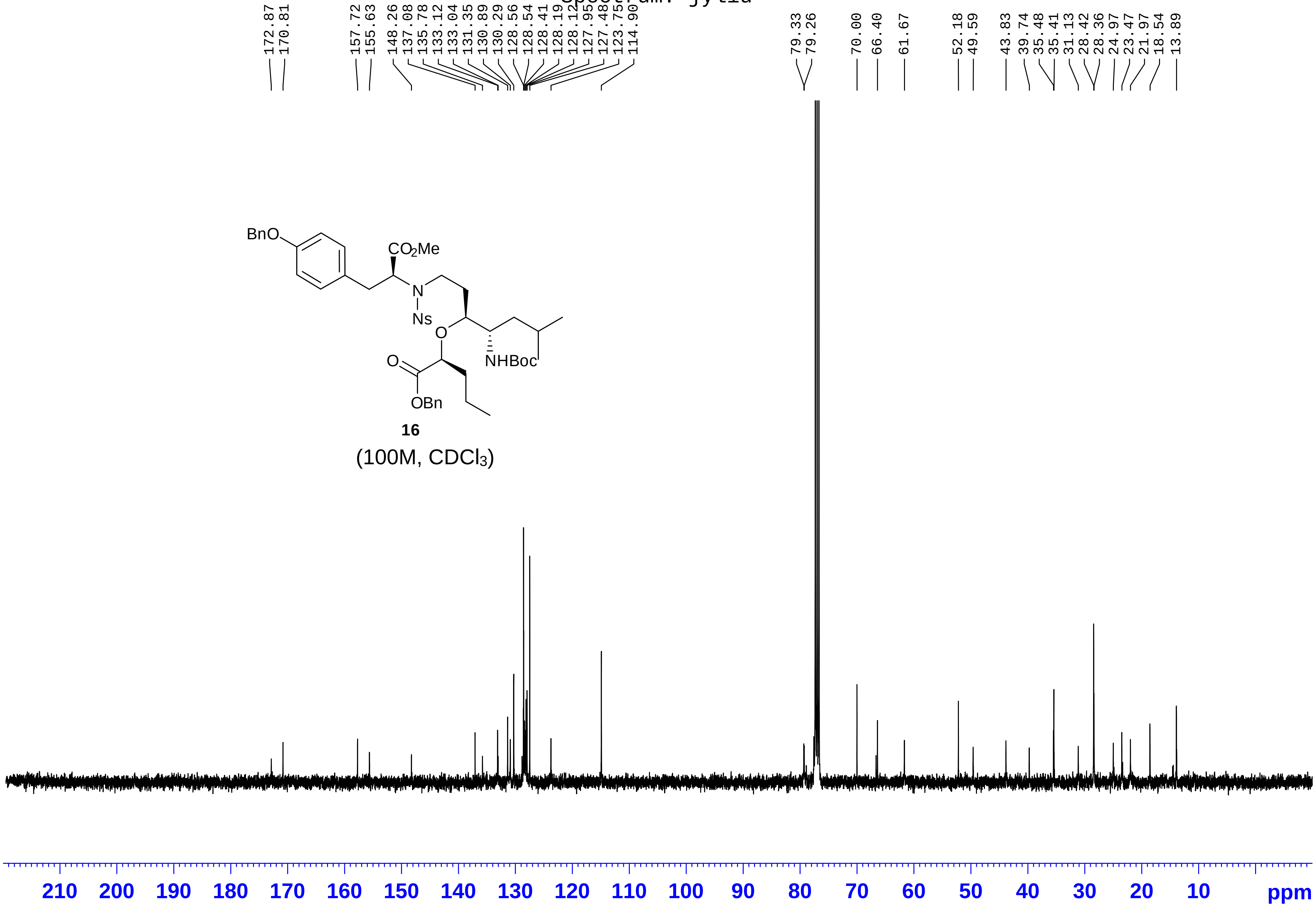

16

(100M, $CDCl_3$)

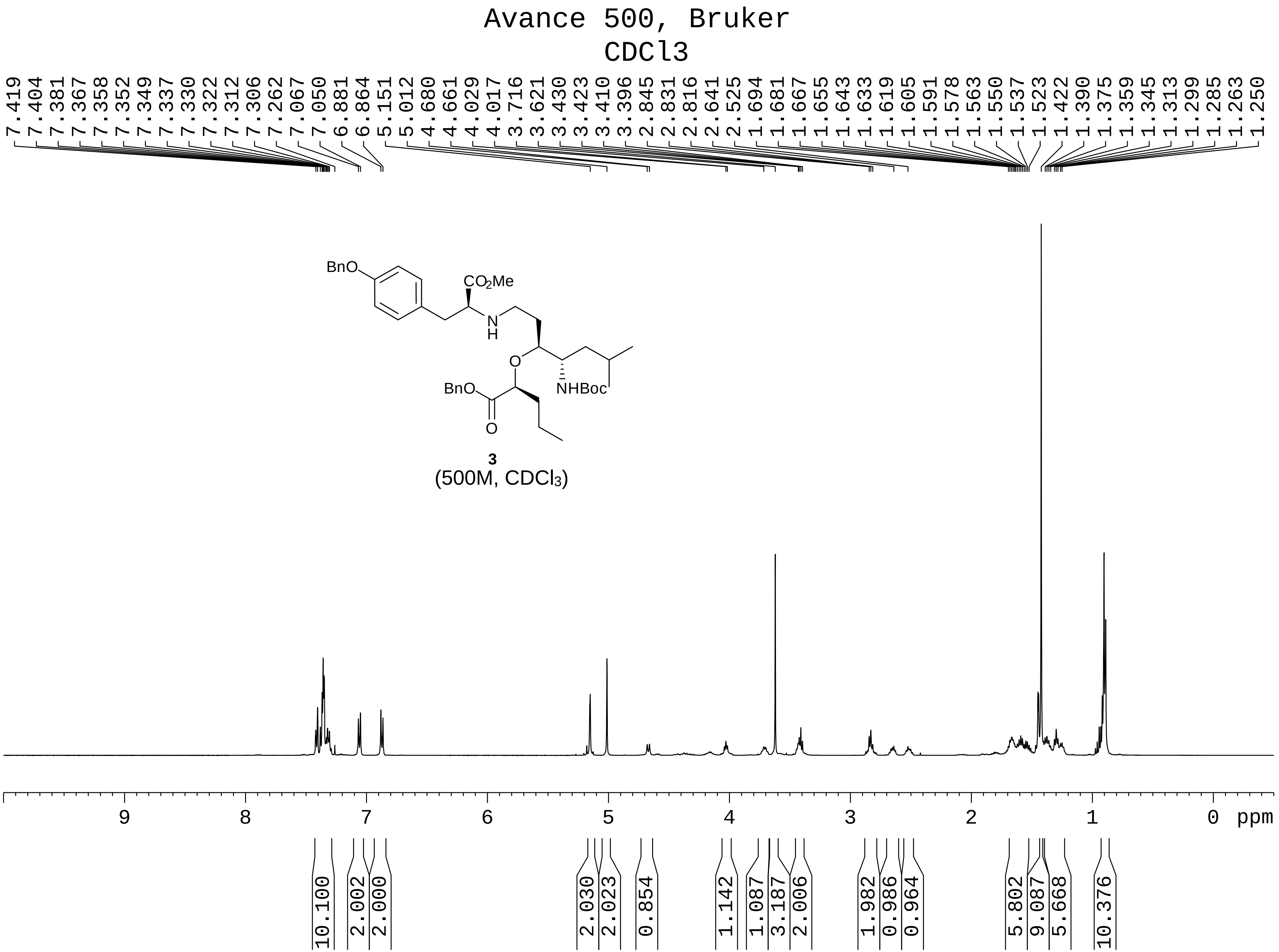
Avance 500, Bruker
CDCl3
7.419 7.404 7.381 7.367 7.358 7.352 7.349 7.337 7.330 7.322 7.312 7.306 7.262 7.067 7.050 6.881 6.864 5.151 5.012 4.680 4.661 4.029 4.017 3.716 3.621 3.430 3.423 3.410 3.396 2.845 2.831 2.816 2.641 2.525 1.694 1.681 1.667 1.655 1.643 1.633 1.619 1.605 1.591 1.578 1.563 1.550 1.537 1.523 1.422 1.390 1.375 1.359 1.345 1.313 1.299 1.285 1.263 1.250
BnO
CO2Me
N
H
O
BnO
O
NHBoc
3
(500M, CDCl3)
9 8 7 6 5 4 3 2 1 0 ppm
10.100 2.002 2.000 2.030 2.023 0.854 1.142 1.087 3.187 2.006 1.982 0.986 0.964 5.802 9.087 5.668 10.376

Avance 500, Bruker, SZPKU
solvent:CDCl3

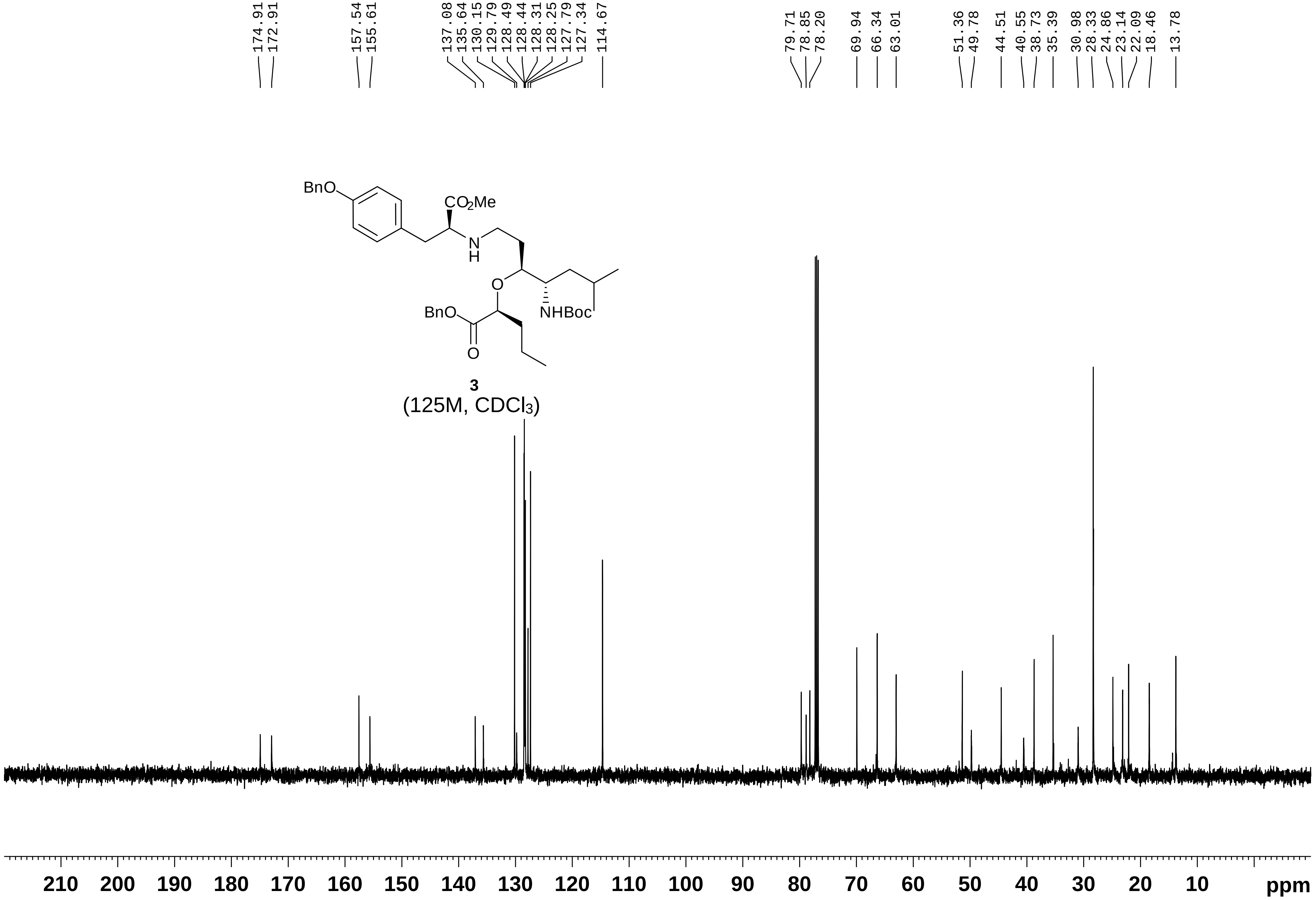

3
(125M, $CDCl_3$)

SZPKU Bruker 400
Solvent: MeOD

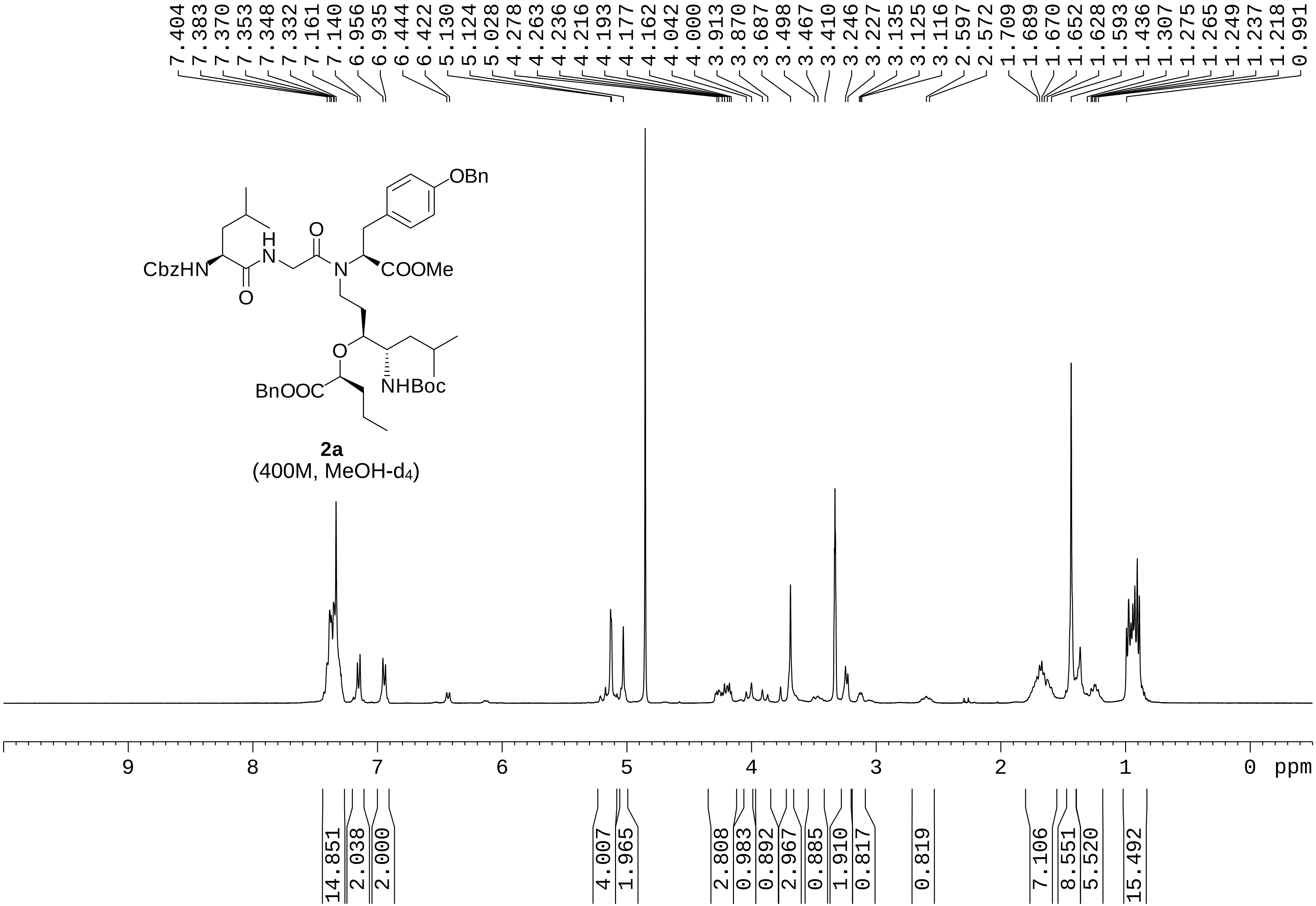

SZPKU Bruker 400
Solvent MeOD

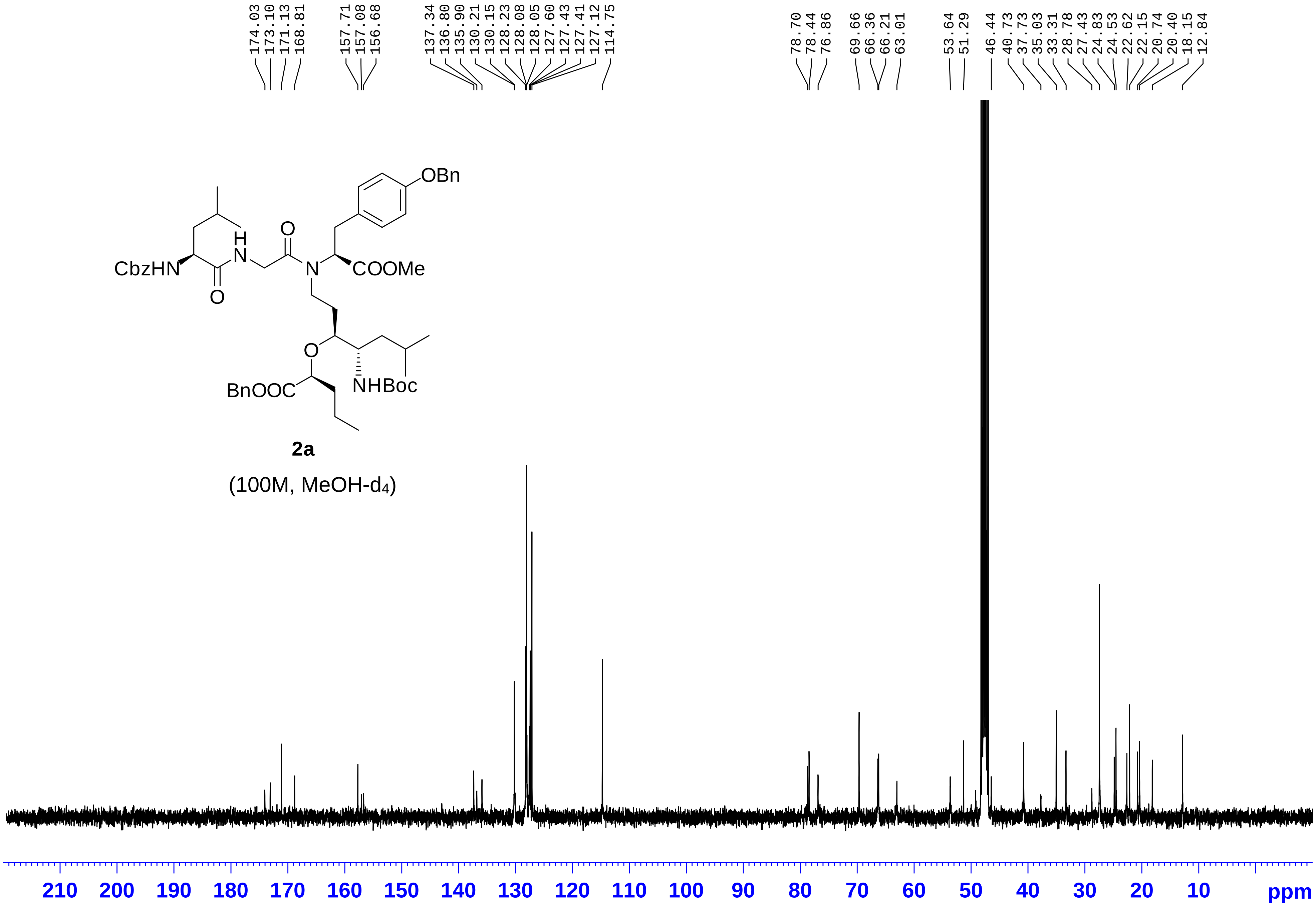

Avance 500, Bruker
solvent:MeOD

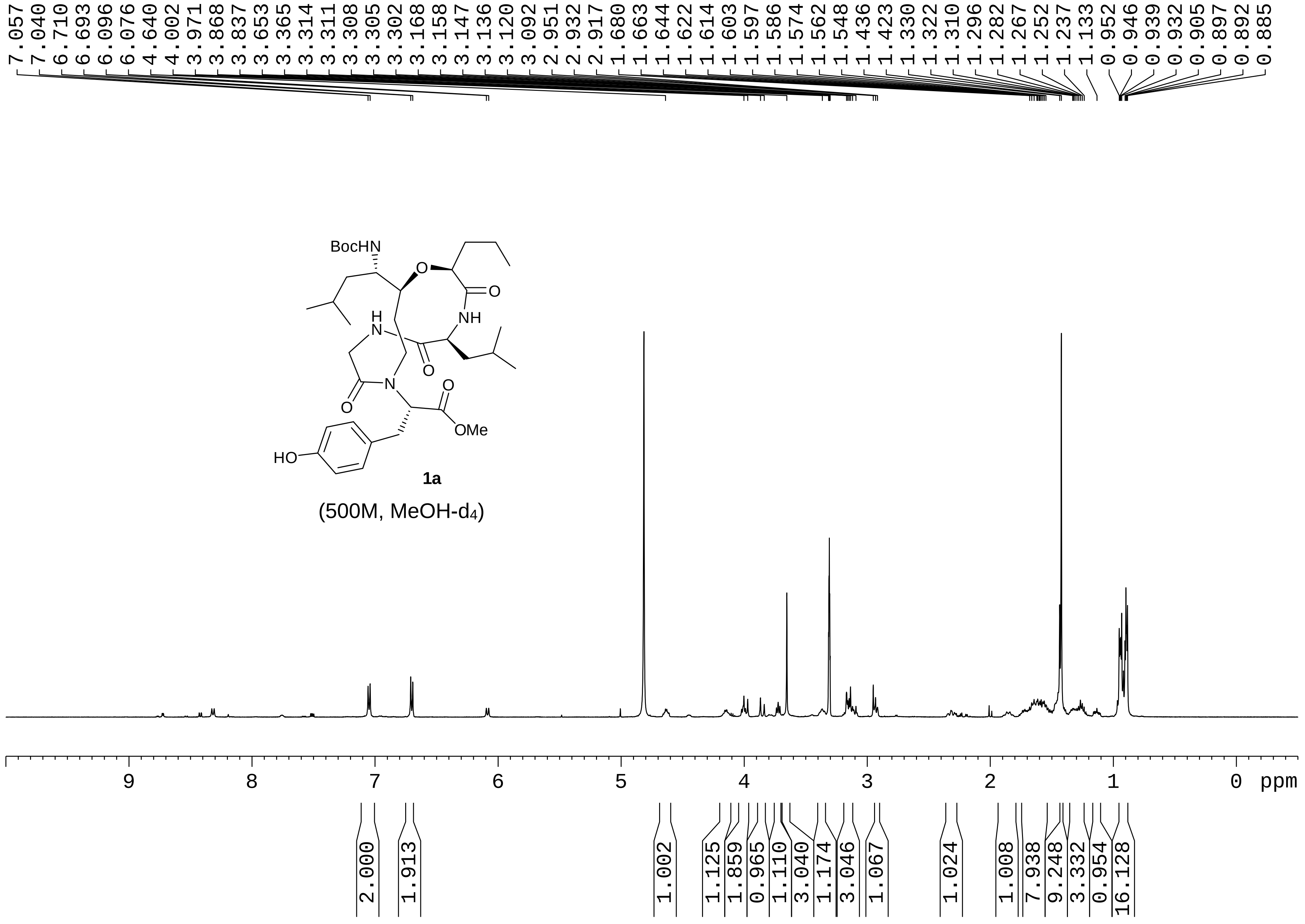

Avance 500, Bruker, SZPKU
solvent:MeOD

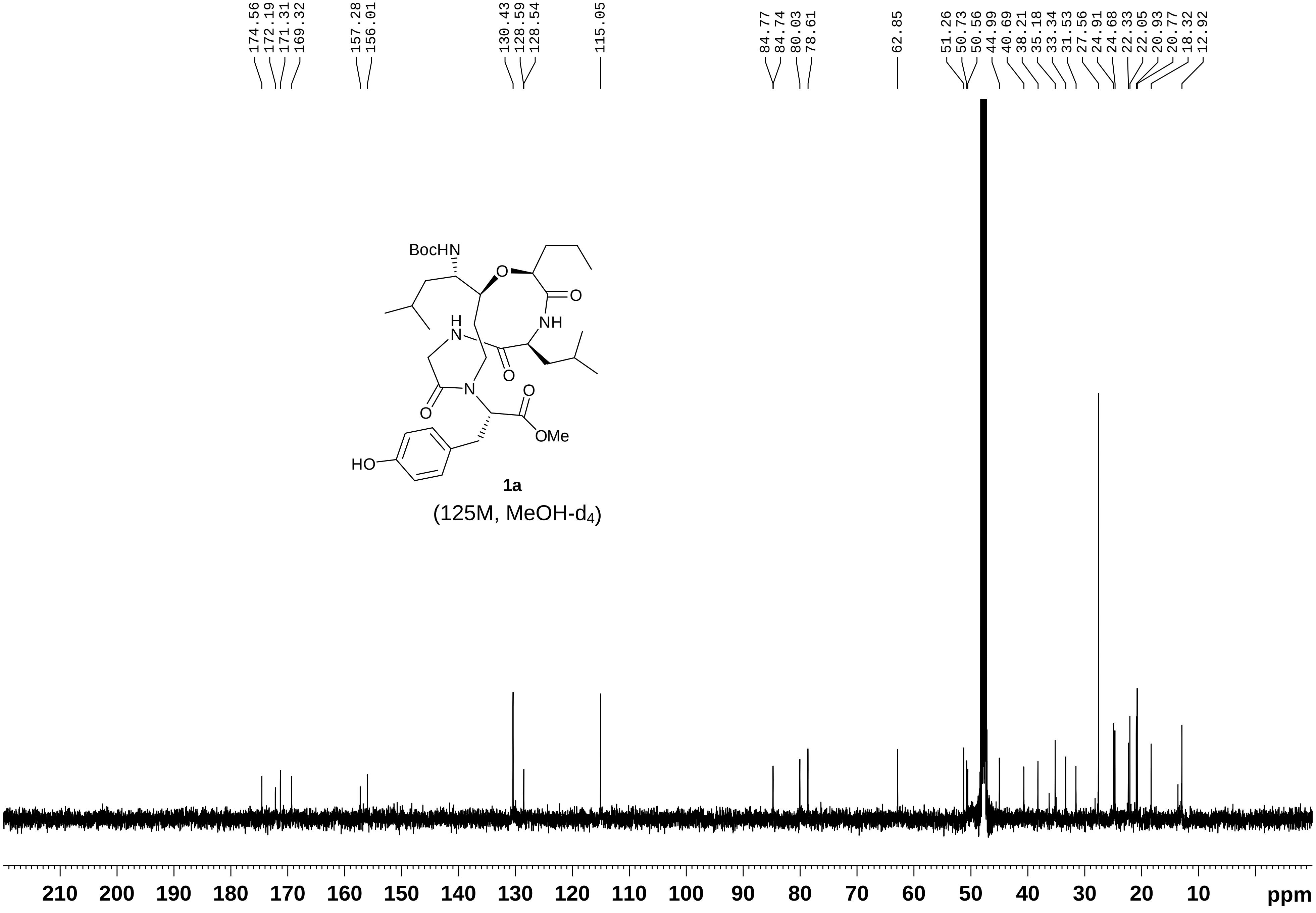

SZPKU Bruker 400
Solvent: MeOD

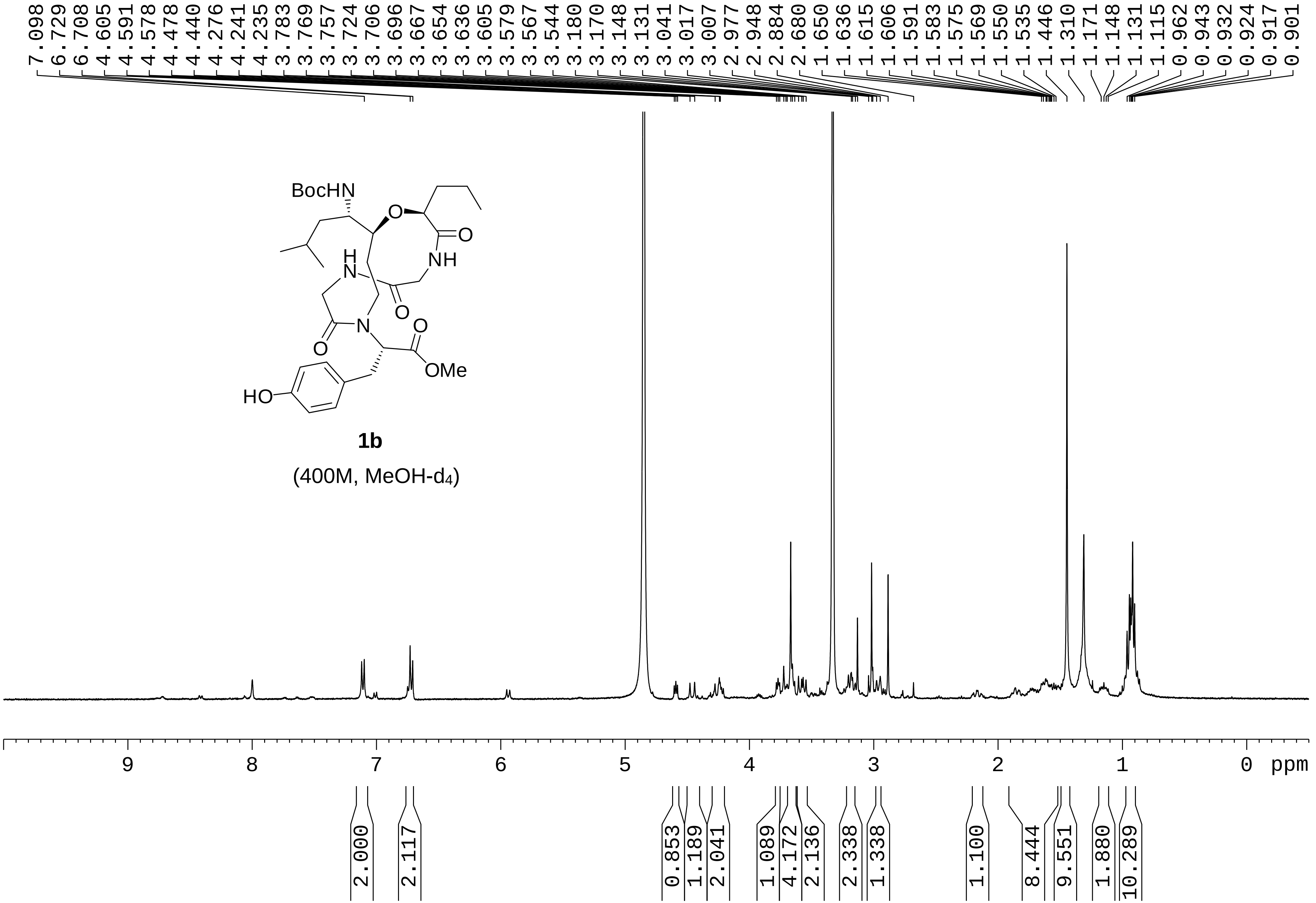

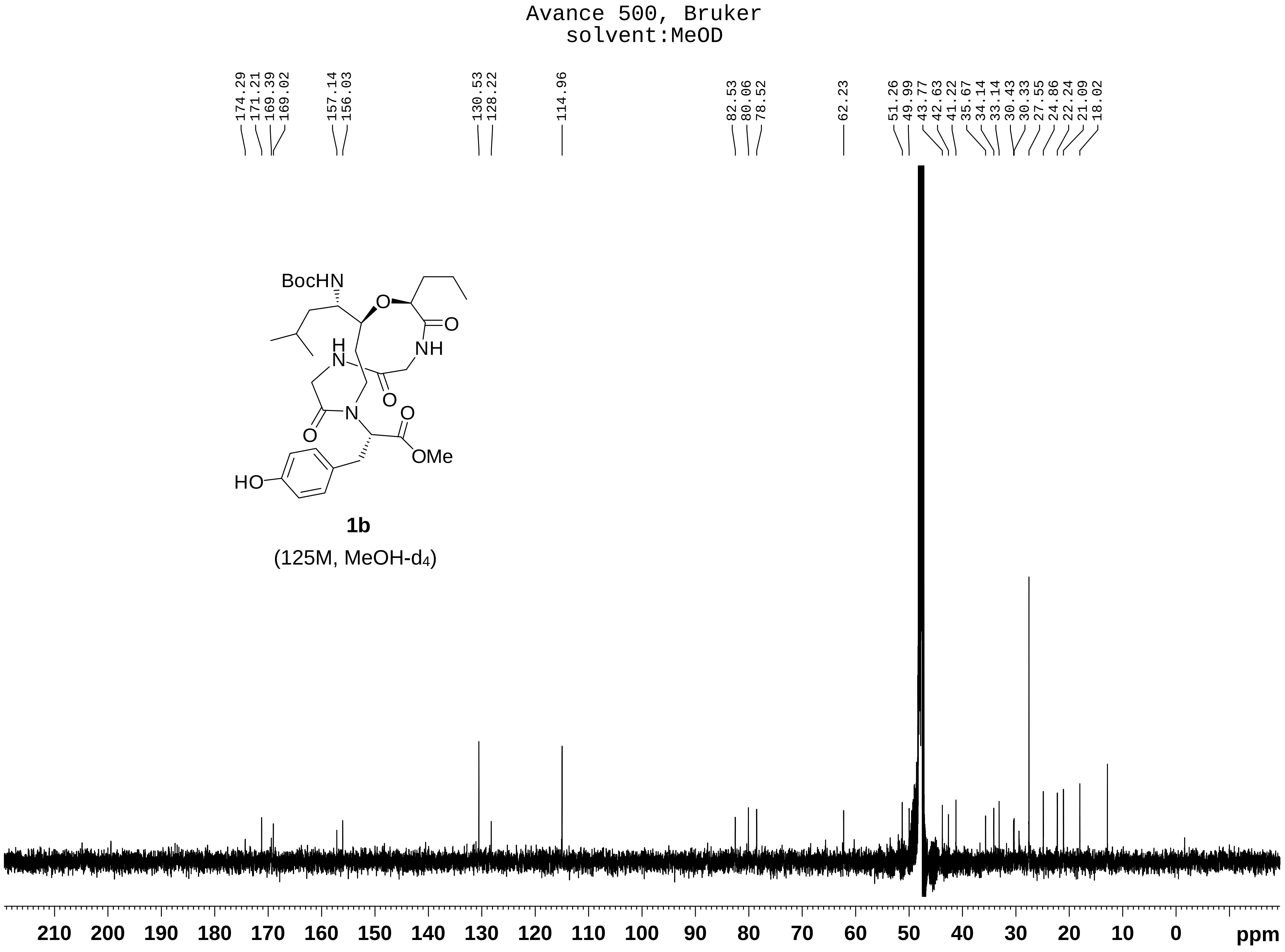
Avance 500, Bruker
solvent:MeOD
174.29 171.21 169.39 169.02 157.14 156.03 130.53 128.22 114.96 82.53 80.06 78.52 62.23 51.26 49.99 43.77 42.63 41.22 35.67 34.14 33.14 30.43 30.33 27.55 24.86 22.24 21.09 18.02
BocHN
O
O
NH
H
N
O
N
O
O
OMe
HO
1b
(125M, MeOH-d4)
210 200 190 180 170 160 150 140 130 120 110 100 90 80 70 60 50 40 30 20 10 0 ppm

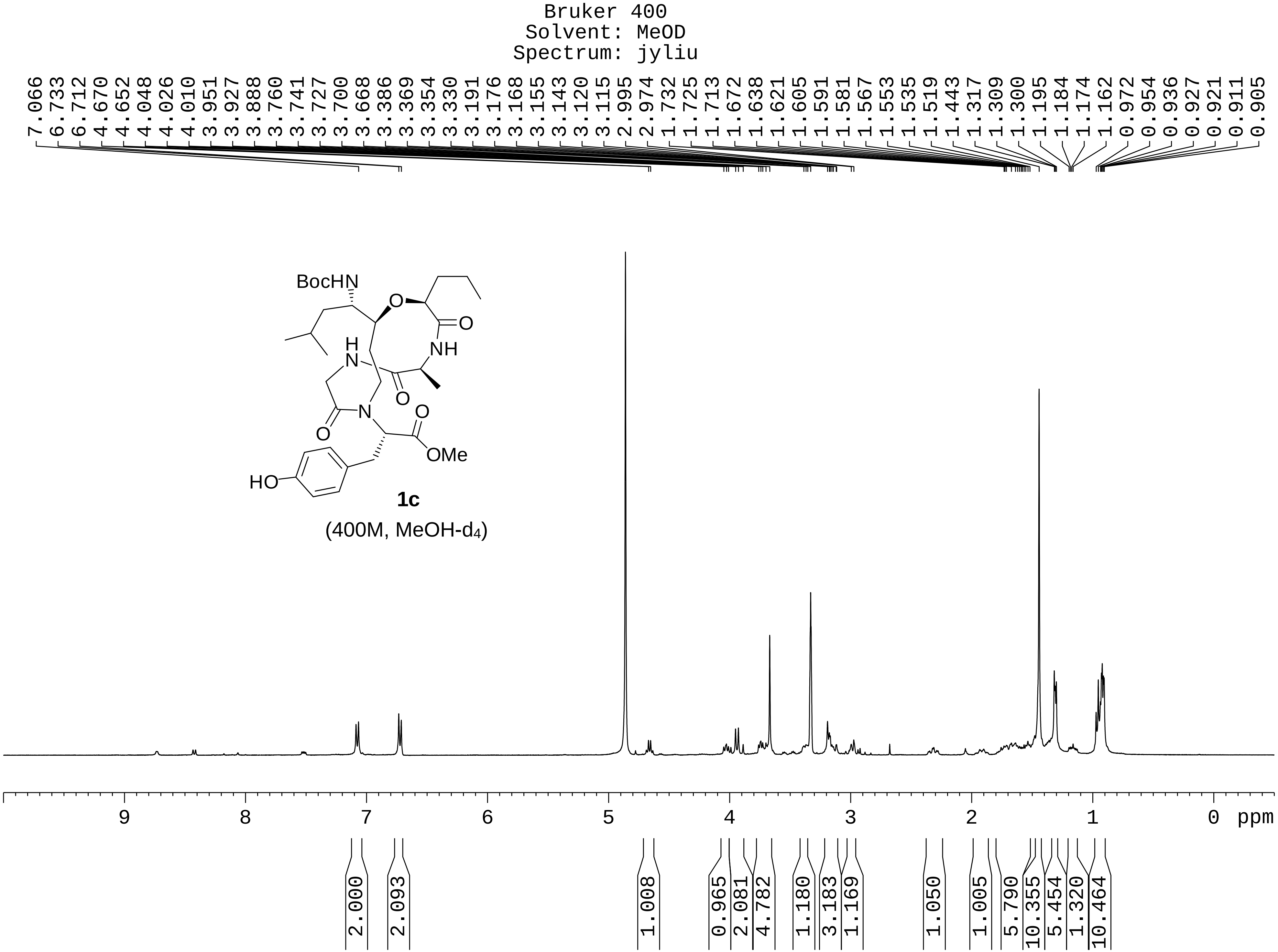
Bruker 400
Solvent: MeOD
Spectrum: jyliu
7.066 6.733 6.712 4.670 4.652 4.048 4.026 4.010 3.951 3.927 3.888 3.760 3.741 3.727 3.700 3.668 3.386 3.369 3.354 3.330 3.191 3.176 3.168 3.155 3.143 3.120 3.115 2.995 2.974 1.732 1.725 1.713 1.672 1.638 1.621 1.605 1.591 1.581 1.567 1.553 1.535 1.519 1.443 1.317 1.309 1.300 1.195 1.184 1.174 1.162 0.972 0.954 0.936 0.927 0.921 0.911 0.905
BocHN
O
O
NH
H
N
O
N
O
O
OMe
HO
1c
(400M, MeOH-d4)
9 8 7 6 5 4 3 2 1 0 ppm
2.000 2.093 1.008 0.965 2.081 4.782 1.180 3.183 1.169 1.050 1.005 5.790 10.355 5.454 1.320 10.464

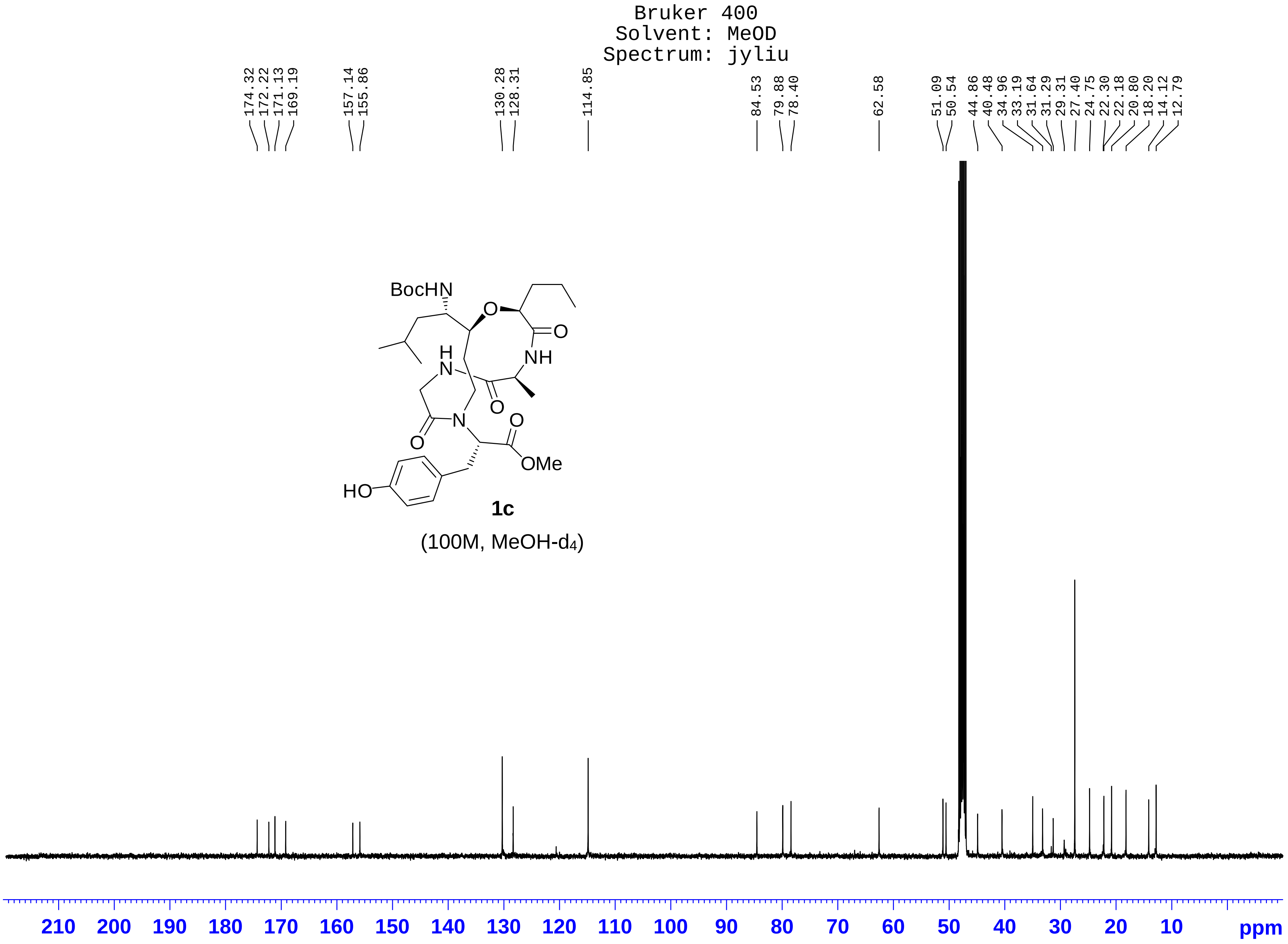
Bruker 400
Solvent: MeOD
Spectrum: jyliu
174.32
172.22
171.13
169.19
157.14
155.86
130.28
128.31
114.85
84.53
79.88
78.40
62.58
51.09
50.54
44.86
40.48
34.96
33.19
31.64
31.29
29.31
27.40
24.75
22.30
22.18
20.80
18.20
14.12
12.79
BocHN
O
O
NH
H
N
O
N
O
O
OMe
HO
1c
(100M, MeOH-d4)
210
200
190
180
170
160
150
140
130
120
110
100
90
80
70
60
50
40
30
20
10
ppm

Avance 500, Bruker
solvent:MeOD

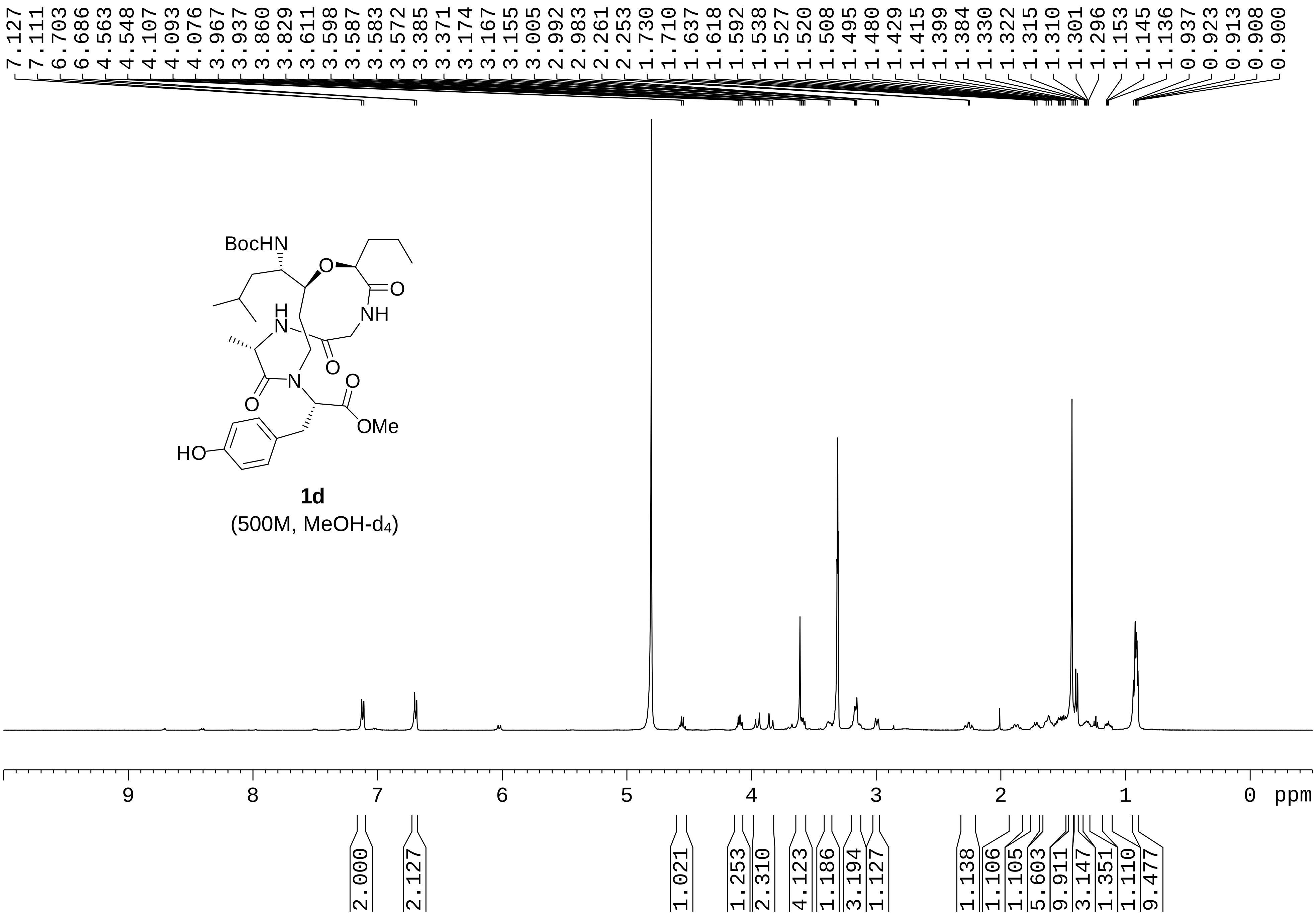

1d
(500M, MeOH-d$_4$)

Avance 500, Bruker
solvent:MeOD
spectrum:jyliu

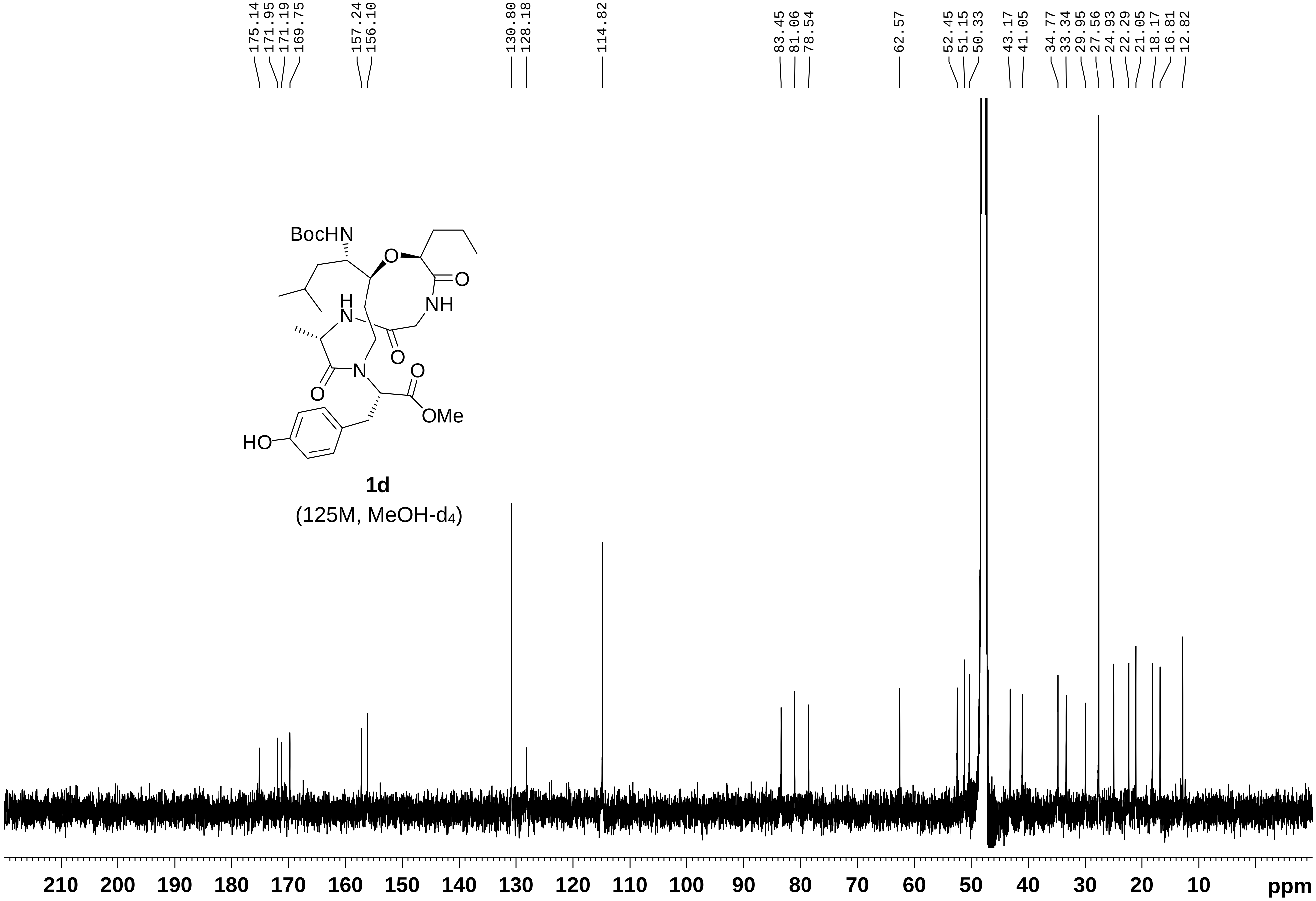

Bruker 400
Solvent: MeOD

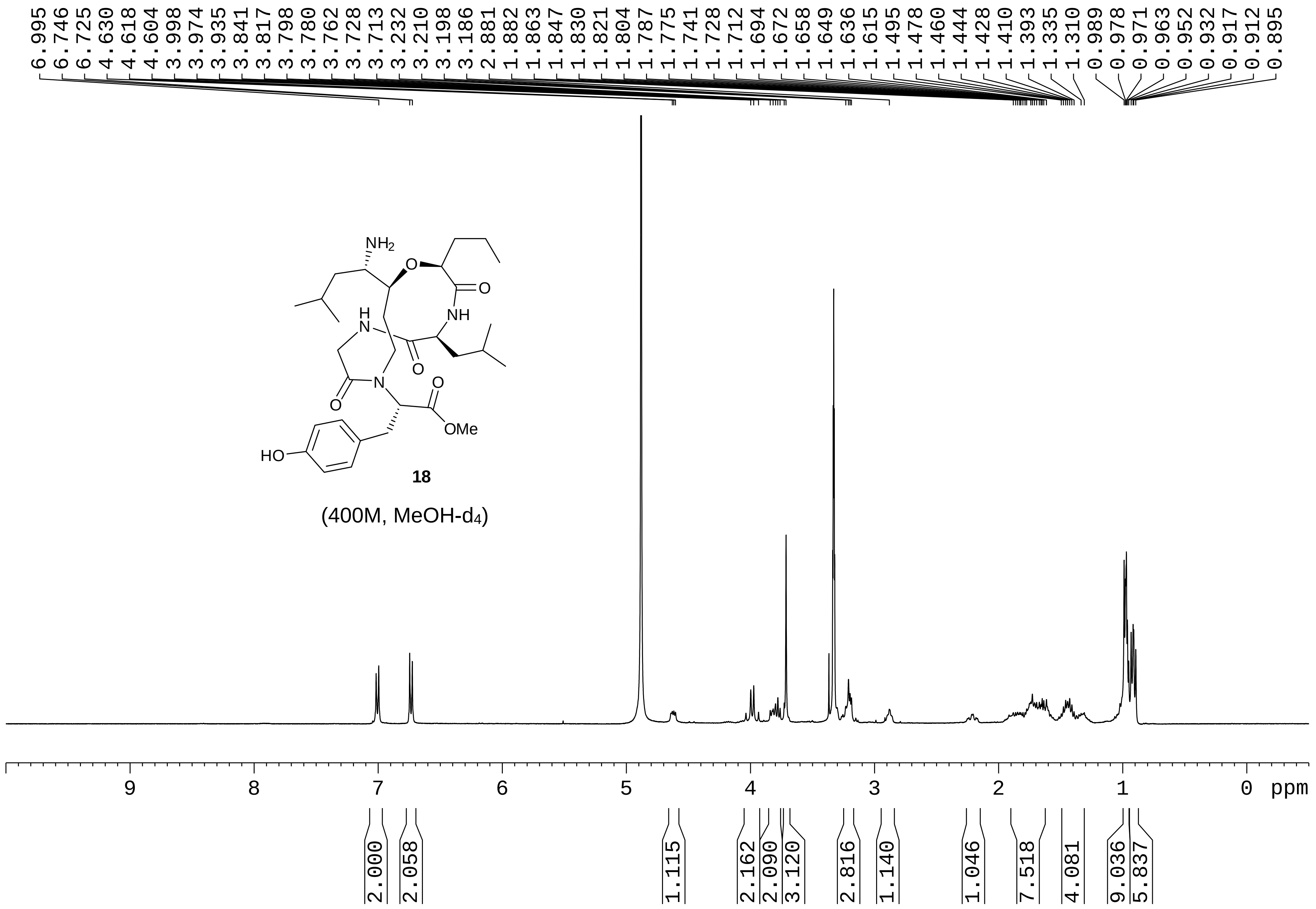

SZPKU Bruker 400
Solvent MeOD

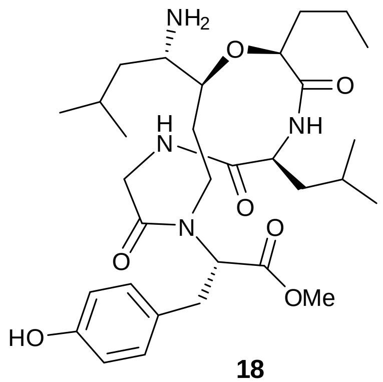

18

(100M, MeOH-d_4)

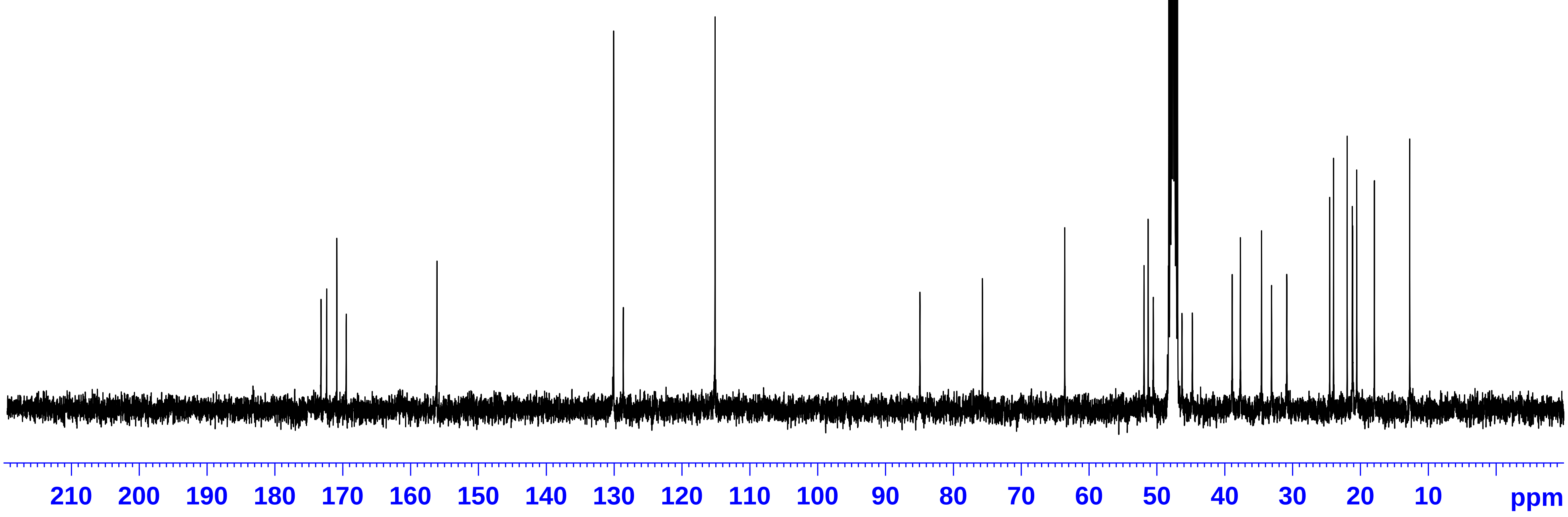

Supplement: Supplementary file 1 [file molecules-28-00780-s001.zip › molecules-2071300-supplementary.pdf]
